# Combined Detection of Intracellular and Extracellular Antibiotic Resistance Genes in Municipal Wastewater—Sample Preparation Methods and Storage Stability

**DOI:** 10.3390/microorganisms14102316

**Published:** 2026-10-10

**Authors:** Sarah Boeker-Haeusser, Hanna Wrede, Claudia Klümper, Silvio Beier

**Affiliations:** 1 Hamm-Lippstadt University of Applied Sciences, 59063 Hamm, Germany; sarah.haeusser@hshl.de (S.B.-H.);; 2 Bauhaus-Institute for Infrastructure Solutions (b.is), Bauhaus University Weimar, 99423 Weimar, Germany

**Keywords:** antimicrobial resistance, antibiotic resistance genes, wastewater, wastewater monitoring, extracellular DNA, intracellular DNA, PCR, sample preparation, sample storage

## Abstract

This study addresses the need for reliable methods in wastewater-based antimicrobial resistance monitoring by comparing three sample preparation approaches for analyzing intracellular DNA (iDNA) and extracellular DNA (exDNA) in raw wastewater: a filtration-based method, a centrifugation-based method, and a combination of the two (combined). We evaluated method repeatability, operational time, usability, consumable costs, qualitative workflow characteristics, and descriptive recovery. Selected antibiotic resistance genes (ARGs) (*sul1*, *bla*_OXA48_ group, *bla*_VIM1_ group, *vanA*, *tetW*, *QnrS1*) and the mobile genetic element *intI1* were quantified by digital PCR. Most target signals occurred in the iDNA-enriched fraction (>98%). Filtration and the combined method received higher usability scores than centrifugation, whereas the combined workflow required approximately 15% higher consumable costs. Storage effects were target-, fraction-, and temperature-dependent. No common holding time satisfied the complete two-sided operational acceptance criteria. Considering decreases in relative recovery alone, storage at 5 °C for up to six days did not reduce any mean recovery below its target- and fraction-specific lower limit. Storage at −20 °C produced decreases in iDNA recovery after 24 h. These findings offer practical guidance for PCR-based AMR monitoring in wastewater.

## 1. Introduction

Antimicrobial resistance (AMR) represents one of the greatest risks to human health. According to current estimates, over 133,000 people across Europe die each year as a direct result of antibiotic-resistant pathogens; approximately half a million additional deaths are associated with these pathogens [1]. Environmental monitoring of AMR is relevant because aquatic systems can function as reservoirs and transmission pathways for resistant microorganisms and resistance genes, and systematic wastewater surveillance complements clinical and veterinary monitoring by enabling the identification of trends and hotspots within the One Health framework [2]. AMR refers to the general ability of microorganisms to evade the effects of antimicrobial agents. In the bacterial context, AMR usually manifests as antibiotic resistance and, phenotypically, as antibiotic-resistant bacteria (ARB). Antibiotic resistance genes (ARGs) are gene sequences that enable bacteria to survive antibiotic exposure and form the genetic basis of AMR by encoding diverse molecular mechanisms of resistance. Along with antibiotics, ARB and ARGs are discharged into wastewater systems [3]. Wastewater treatment plants (WWTPs) are considered likely hotspots for the spread of antibiotic resistance, as they combine high DNA loads from lysed cells, dense and genetically diverse microbial communities in raw and treated wastewater, as well as in sewage sludge, and sustained selection pressure from antibiotics and other stress factors, creating ideal conditions for the reproduction of ARBs and the horizontal gene transfer (HGT) of ARGs [3,4,5,6,7,8,9,10]. To address these issues, the revised EU Urban Wastewater Treatment Directive (Directive (EU) 2024/3019) establishes a coordinated framework for urban wastewater surveillance in Article 17 and mandates the monitoring of AMR in agglomerations of ≥100,000 population equivalents [11].

ARGs are detected in both the intracellular DNA (iDNA) and extracellular DNA (exDNA) fractions of environmental samples. The exDNA fraction originates from bacterial cell lysis and active secretion by living bacteria. It often contains mobile genetic elements (MGEs), such as plasmids, integrons, and transposons, as well as free-floating DNA [12,13]. Bacteria can acquire exDNA containing ARGs from wastewater through conjugation (direct cell-to-cell contact), transformation, transduction, or membrane vesicle-assisted transformation. These modes of HGT enable bacteria to rapidly acquire new genetic traits, such as antibiotic resistance, from their environment [5,14,15].

Studies have already shown that ARGs, including several high-risk ARGs, are present in the iDNA and exDNA fractions of treated and raw wastewater [12,16]. Although ARGs are typically more abundant in the iDNA fraction, and exDNA concentrations in raw wastewater are generally low, multiple studies emphasize the importance of including exDNA in addition to iDNA for comprehensive ARG monitoring [5,8,12,17,18,19,20]. ExDNA can represent a substantial fraction of total DNA in treated wastewater and may persist or increase relative to iDNA throughout wastewater treatment processes [8,16,17].

For these reasons, molecular biological methods that detect ARGs and differentiate between exDNA and iDNA fractions are increasingly important for analyzing wastewater and other environmental samples [5,21]. As iDNA is operationally associated with the cellular/particulate fractions, most studies to date have used filter-based methods [4,22,23,24]. Separating the cell-free filtrate/liquid fraction by filtration or centrifugation enables exDNA detection, although adsorption to particles may occur [5]. Although multiple studies have evaluated the prevalence and fate of ARGs in iDNA and exDNA fractions in wastewater with PCR-based methods and metagenomic sequencing [8,14], studies investigating practical solutions for the combined detection of iDNA and exDNA are rare [5,14].

In addition to knowledge of the distribution of individual DNA fractions within a sample, another crucial factor for reliable characterization of ARGs in wastewater samples is their stability during sample storage. Only when such stability is assured can monitoring data be relied upon for risk assessment. Only a few studies to date have addressed the influence of storage conditions (time and temperature) on the stability of parallel analysis of iDNA and exDNA prior to extraction [17,25]. Studies have shown that storage conditions affect total DNA and iDNA and that both preservation methods (e.g., ethanol fixation, freezing) and prompt processing are crucial for ensuring DNA integrity and maintaining representative resistance profiles [26,27]. To date, no direct comparative analysis of the stability of intracellular and extracellular ARGs in raw wastewater samples as a function of storage duration has been conducted.

Regarding the revised EU Urban Wastewater Treatment Directive (Directive (EU) 2024/3019), both aspects, (1) appropriate sample preparation methods to differentiate between exDNA and iDNA, as well as (2) stability of ARGs in raw wastewater, are highly relevant for practical implementation in the water sector [11]. Thus, the objective of the present study is twofold: (1) The first objective was to conduct an exploratory pilot comparison of three wastewater sample preparation methods: filtration, centrifugation, and a combined method, for the parallel analysis of iDNA- and exDNA-enriched DNA fractions. The ARGs *sul1*, *bla*_OXA-48_ group, *bla*_VIM-1_ group, *vanA*, *tetW*, and *qnrS1*, together with the mobile genetic element *intI1*, were quantified in both fractions. Using a homogenized 24-h composite influent wastewater sample, we characterized the methods’ repeatability, descriptive total-process recovery, operational time, usability, consumable costs, and qualitative workflow and resource requirements. This pilot study aimed to identify matrix-specific operational strengths and limitations of the three methods and to provide a basis for selecting workflows for subsequent validation. In addition, (2) we conducted a stability test to assess the influence of storage time and storage temperature on intracellular and extracellular ARGs in a raw wastewater sample.

## 2. Materials and Methods

**Figure 1:**
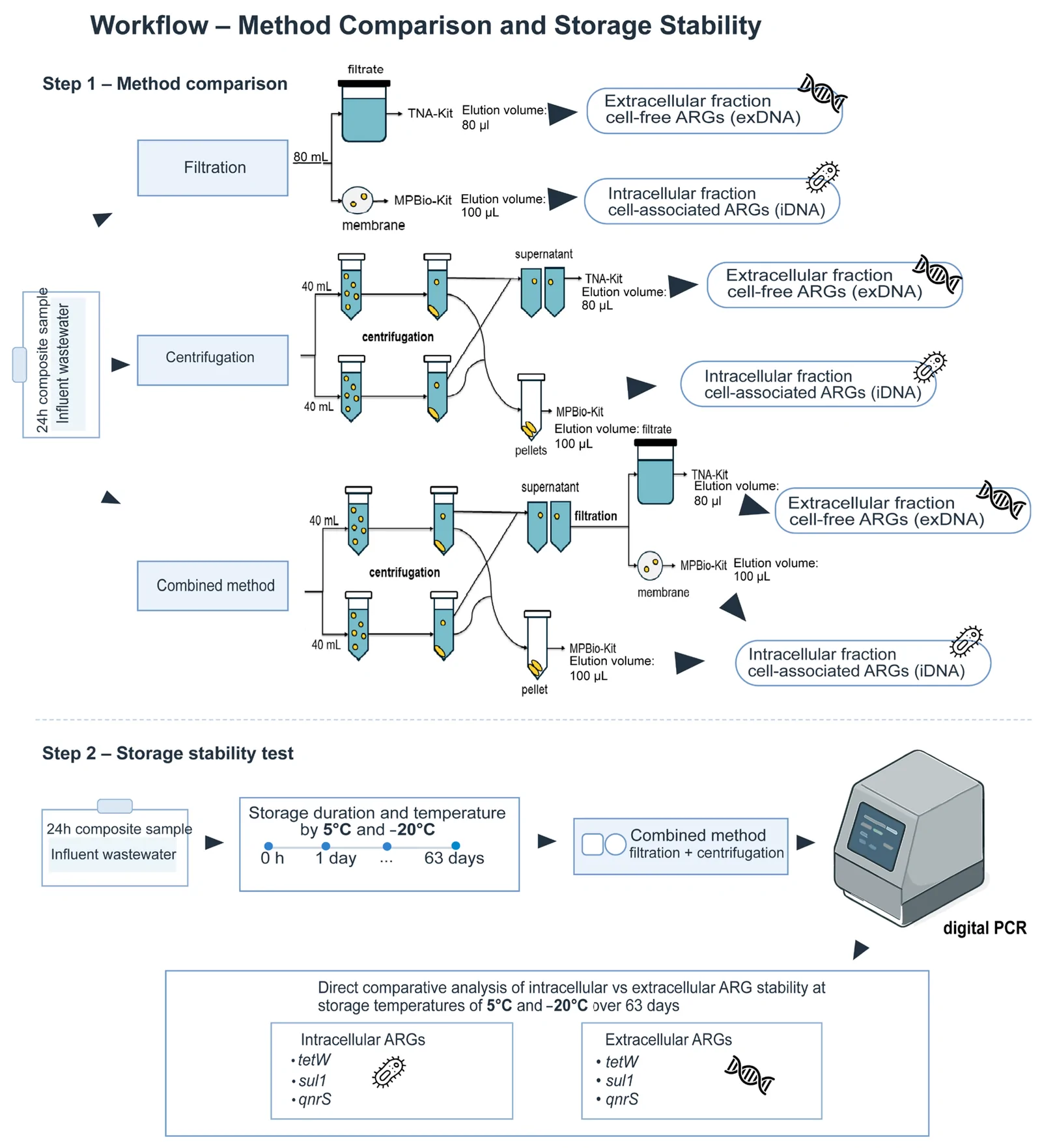
Workflow for method comparison and assessment of storage stability.

Figure 1 illustrates our workflow for the method comparison and storage stability test. In the first step, an in-house laboratory method comparison was conducted among a filtration-based method, a centrifugation-based method, and a combined approach (filtration and centrifugation).

For the method comparison, the wastewater extracts were analyzed for the following targets: *bla*_OXA48_ group, *bla*_VIM1_ group, *sul1*, *qnrS1*, *vanA*, *tetW*, and *intl1*. To determine the recovery rate, the unspiked and spiked wastewater extracts were analyzed for the target *E. coli*.

To assess the stability of intracellular and extracellular ARGs in raw wastewater over the storage period, we analyzed *sul1*, *tetW*, and *qnrS1* using dPCR. After comparing the methods, we conducted a storage stability test using the combined approach.

In this study, iDNA and exDNA refer to operationally defined fractions obtained using the respective sample preparation methods. The iDNA-enriched fraction comprised cell-associated material retained by filtration and/or recovered by centrifugation, whereas the corresponding filtrate or supernatant was used to recover the exDNA-enriched fraction. However, this separation does not represent an absolute distinction between intracellular and extracellular DNA. Cell-associated DNA may originate from viable, viable-but-nonculturable, or dead cells. In contrast, cell-free DNA can occur as freely dissolved DNA or be associated with particles, membrane vesicles, or bacteriophages [5]. Therefore, throughout this study, these resulting fractions are referred to as iDNA-enriched and exDNA-enriched fractions.

We selected the targets analyzed in this study for their relevance to wastewater-based monitoring, their association with anthropogenic inputs, and their clinical resistance [23,28]. *tet(W)* is commonly associated with wastewater sources [29], while *intI1* and *sul1* are linked to class 1 integrons [30] and have been proposed as indicators of antibiotic resistance and anthropogenic pollution [23,28]. Furthermore, *intI1*, *sul1*, and *vanA* are among the most frequently investigated targets in wastewater-based ARG monitoring [23]. A Europe-wide study on AMR in surface waters and wastewater treatment plant influents also supported the selection of *intI1*, *sul1*, *tet(W)*, and *vanA* [31]. The relevance of the selected target panel is further supported by the WHO pilot guidance on wastewater and environmental surveillance for AMR. The WHO identifies carbapenem-resistant *Enterobacterales* as a critical-priority pathogen–antimicrobial combination and classifies carbapenem-resistant *Pseudomonas aeruginosa*, vancomycin-resistant *Enterococcus faecium*, and several fluoroquinolone-resistant bacterial pathogens as high-priority combinations. Although these priorities were not developed specifically for wastewater and environmental surveillance, the WHO considers them a useful starting point for aligning wastewater-based AMR monitoring with broader clinical and public health surveillance priorities [32]. Accordingly, *bla*_OXA-48_-like, *bla*_VIM_, *vanA*, and *qnrS1* are molecular determinants of resistance associated with several internationally prioritized resistance phenotypes. The targets *sul1*, *tet(W)*, and *qnrS1* were selected for the storage-stability experiments because they occur at comparatively high concentrations in raw wastewater, enabling reliable quantification over the investigated storage period. In addition, the targets represent different genetic localisations: *sul1* and *qnrS1* are frequently associated with plasmids or other mobile genetic elements, whereas *tet(W)* is often chromosomally encoded [33].

### 2.1. Method Comparison

We used the following domains in Table 1 to evaluate the different methods:

Wastewater sampling and transport

A 24-h composite, volume-proportional, five-liter influent wastewater sample was collected from the influent of a municipal wastewater treatment plant (population equivalents of 252,000 (EW)) in Germany during the night from Monday to Tuesday in July 2024.

The following standard physico-chemical parameters were analyzed on site at the WWTP according to generally accepted standard methods (ISO standards): ammonium [34], total nitrogen [35], and chemical oxygen demand [36].

The wastewater sample was transported to the laboratory in a cooled (5 ± 2 °C) Styrofoam box and processed (homogenization and subsampling) immediately upon arrival. Homogenization was performed using a QMR 25 homogenizer (Behr Labor-Technik GmbH, Düsseldorf, Germany). The sample was analyzed in six process replicates for each method, with a volume of 80 mL: three process replicates with an *E. coli* spike and three without.

To determine the recovery rate, the 80 mL process replicates without a spike were aliquoted whilst the sample was being stirred. Then the remaining wastewater sample (2.8 L) was spiked accordingly with 4.2 mL of an *E. coli* spike suspension (corrected Conc. 5.1 × 10^9^ GC/mL) (Supplement Preparation of the *E. coli* spike and Table S7). Three process replicates, each containing 80 mL, were prepared for each method using the spiked wastewater sample. DNA associated with the membrane and pellet fractions was extracted and eluted in 100 µL, whereas DNA from the filtrate fraction was eluted in 80 µL. We back-calculated fraction-specific concentrations to the original wastewater volume. For whole-process recovery assessment, we summed the concentrations determined in the corresponding iDNA- and exDNA-enriched fractions. As a positive control, we used an autoclaved drinking water sample spiked with *E. coli*.

Wastewater nucleic acid extraction and concentration

The following brief descriptions outline the general process for each method of sample preparation and nucleic acid extraction for each process replicate (80 mL). Figure 1 shows the method details [17,37].

Nuclease-free water (Promega GmbH, Walldorf, Germany) is used as a negative extraction control.

Filtration:

For the iDNA extraction, the samples are filtered through a 0.22 µm, 47 mm, polycarbonate membrane (Whatman^TM^, Nuclepore^TM^ Track-etched, Cytiva, Marlborough, MA 01752, USA) using a pressure filtration unit 200 mL (Sartorius Pressure Filtration Unit Stainless Steel, Sartorius AG, Göttingen, Germany) to separate the solid fraction from the filter. The iDNA is extracted from the filter using the FastDNA^TM^ SPIN Kit for Soil (MP Biomedicals Germany GmbH, Eschwege, Germany) with FastPrep-24^TM^ 5G Homogenizer (MP Biomedicals Germany GmbH, Eschwege, Germany) according to the manufacturer’s instructions, with a final eluate volume of 100 µL. The filtrate is collected in a sterile container, and the exDNA-enriched fraction is extracted using the Maxwell RSC Enviro Total Nucleic Acid Kit (Promega GmbH, Walldorf, Germany) with the Maxwell^®^ RSC Instrument (Promega GmbH, Walldorf, Germany) according to the manufacturer’s instructions, for a final eluate volume of 80 µL.

Centrifugation:

For iDNA extraction, centrifuge the samples for 20 min at 10.000 g and 4 °C to separate the cells. The iDNA is extracted from the pellet using the FastDNA^TM^ SPIN Kit for Soil (MP Biomedicals Germany GmbH, Eschwege, Germany) with FastPrep-24^TM^ 5G Homogenizer (MP Biomedicals Germany GmbH, Eschwege, Germany) according to the manufacturer’s instructions, with a final eluate volume of 100 µL. The supernatant is transferred to sterile tubes for exDNA extraction using the Maxwell RSC Enviro Total Nucleic Acid Kit (Promega GmbH, Walldorf, Germany) with the Maxwell^®^ RSC Instrument (Promega GmbH, Walldorf, Germany) according to the manufacturer’s instructions, for a final eluate volume of 80 µL.

Combined method:

The procedure was as described in the “Centrifugation” method, except that the supernatant after centrifugation was then processed using the “filtration” method. Pellets and membrane were used to extract iDNA using the FastDNA^TM^ SPIN Kit for Soil (MP Biomedicals Germany GmbH, Eschwege, Germany) with a FastPrep-24^TM^ 5G Homogenizer (MP Biomedicals Germany GmbH, Eschwege, Germany) according to the manufacturer’s instructions, with a final eluate volume of 100 µL. The filtrate is collected in a sterile container, and the exDNA-enriched fraction is extracted using the Maxwell RSC Enviro Total Nucleic Acid Kit (Promega GmbH, Walldorf, Germany) with the Maxwell^®^ RSC Instrument (Promega GmbH, Walldorf, Germany) according to the manufacturer’s instructions, for a final eluate volume of 80 µL. For the method comparison, the eluates from the combined method (pellet and membrane) were quantified separately to evaluate the CVs. To determine the overall concentration (GC/mL), the concentrations (GC/mL) of the pellet and the membrane were summed. After the method comparison, for the combined method, the eluates from the pellet and the membrane were physically combined, resulting in a final eluate volume of 200 µL, and prepared for analysis as an iDNA-enriched fraction for dPCR analysis.

The total DNA concentration [µg/mL], A260 nm/A280 nm quotient, and absorbance at 260 nm of the DNA extracts were measured using a NanoDrop spectrophotometer (Eppendorf BioPhotometer D30, Eppendorf SE, Hamburg, Germany).

DNA detection and quantification with digital PCR

For detection and quantification of ARG-specific DNA sequences, we used a digital PCR system (QIAcuity One, 5plex Instrument, QIAGEN GmbH, Hilden, Germany) with QIAcuity Nanoplate 26k (QIAcuity Nanoplate 26k 24-Well, QIAGEN GmbH, Hilden, Germany). Information on the primer-probe assays is provided in the supplement.

The digital PCR assays were set up in a reaction volume of 40 µL, including 10 µL of nucleic acid extract, 10 µL of 4 × QIAcuity Probe PCR Kit (QIAGEN GmbH, Hilden, Germany), 2 µL of 20 × primer/probe per assay, and nuclease-free water. Cycling conditions for the digital PCR assay for detection of *E. coli*, *bla*_OXA48_ group, *bla*_VIM1_ group, *sul1*, *qnrS*, and *vanA* were as follows: polymerase activation at 95 °C for 2 min and 40 cycles of denaturation, annealing/extension at 95 °C/15 s, then 62 °C/60 s, respectively. The cycle conditions for the digital PCR assay for detecting *intl1* and *tetW* were as follows: polymerase activation at 95 °C for 2 min, followed by 45 cycles of denaturation, annealing/extension at 95 °C/15 s, then 62 °C/60 s.

Equation (1) shows how to convert dPCR measurement values [gene copies per mL master mix] (the direct result from the dPCR software (QIAcuity Software Suite 3.2.0.0), calculated using Poisson statistics) into gene copies per milliliter of wastewater.

(1) $$\mathrm{Gene}\;\mathrm{copies}\;\mathrm{per}\;\mathrm{mL}\;\mathrm{wastewater}=C_{\mathrm{dPCR}}\times \frac{V_{\mathrm{dPCR}}}{V_{\mathrm{Template}}}\times \frac{V_{\mathrm{Eluate}}}{V_{\mathrm{Wastewater}}}\times D$$

*C_dPCR_* = *Direct result from the dPCR software* [*gene copies per µL master mix*]

*V_dPCR_* = *Total volume of the prepared dPCR mixture* (*master mix* + *template*) [µL]

*V_Template_* = *Volume of extracted DNA used in the dPCR* [µL]

*V_Eluate_* = *Total volume of extracted DNA after elution* [µL]

*V_Wastewater_* = *Initial volume of wastewater used* (*before concentration*) [mL]

*D* = *Pre* − *dilution factor of the extracted sample* (*if undiluted*: *D* = 1)

All samples were run with one technical replicate and a 1:100, 1:1000, or 1:10,000 dilution. No-template control (NTC) and positive control were also analyzed in each run to exclude contamination and verify proper digital PCR performance. Positive controls used for the ARGs *bla*_OXA48_ group, *bla*_VIM1_ group, *sul1*, *qnrS*, and *vanA* were from Qiagen Microbiota Control (Microbial DNA positive control-V2; QIAGEN GmbH, Hilden, Germany), and positive controls for *tetW* and *intl1* included in the dPCR-Kit (GT-Digital intl1; tetW Assay Kit for the QIAcuity^®^ Digital PCR System, Fort Collins, CO, USA). We determined the Limit of Quantification (LoQ) by quantifying antibiotic-resistant gene copy numbers in technical dPCR replicates across a 1:10 dilution series of DNA-positive control material. A Gaussian distribution was fitted to the proportion of positive technical replicates along the dilution series and used to estimate the 95% LoQ per µl (Figure S1 in the Supplement) [38,39]. We determined the LoQ for the ARGs examined by digital PCR, as shown in Figure S1 in the Supplement. The following values were determined: *bla*_VIM-1_ group: 0.24 GC/µL; *vanA*: 0.14 GC/µL; *bla*_OXA-48_ group: 0.15 GC/µL; *QnrS1*: 0.04 GC/µL; *tetW*: 0.12 GC/µL; *sul1*: 0.36 GC/µL. For replicates that show positive partitions but a concentration < LoQ, set the concentration to ½ LoQ.

### 2.2. Storage Stability

To test storage stability, another five-liter sample was taken as a 24 h composite sample (volume-proportional) from the influent of the municipal WWTP in October 2025. Sampling and homogenization were carried out as described in Section 2.1.

During homogenization, 47 process replicates were taken, each containing 80 mL of wastewater and processed using the combined method. To capture natural variation among process replicates, 11 of the 47 process replicates were extracted immediately for analysis, and the extracts were stored at −80 °C until analysis of the targets *sul1*, *tetW*, and *QnrS1* by dPCR. Of the remaining 36 process replicates, 18 aliquots were stored at 5 °C in the refrigerator, and 18 aliquots were stored at −20 °C in the freezer. As shown in Table 2, aliquots from the 5 °C and −20 °C storage were taken and extracted on 9 measurement days. These extracted aliquots were also stored at −80 °C until analysis by dPCR. The aliquots were stored for up to 63 days.

A common baseline dataset, collected immediately after sample preparation (Measurement point t0), served as a reference for both storage temperatures. The analysis was carried out separately for *sul1*, *tetW* and *qnrS1*, as well as for the iDNA- and exDNA-enriched fractions. Storage-related changes were evaluated in accordance with ISO/TS 5667-25:2022 [40]. Using the arithmetic value assigned to t0 as a reference point, we evaluated the storage-related relative changes for each target and each DNA fraction. The relative recovery (RR) of a single observation (i) at a given storage time (t) was calculated using Equation (2).

(2) $${\mathrm{RR}}_{t,i}\left[\%\right]=\frac{x_{t,i}}{{\bar{x}}_{0}}\times 100$$

*x_t,i_* = *gene concentration per* mL *of replicate* (*i*)

*x*_0_ = *arithmetic mean gene concentration per* mL *at t*0

The mean relative recovery at each measurement point was calculated as in Equation (3):

(3) $$\bar{{\mathrm{RR}}_{t}}=\;\frac{{\bar{x}}_{t}}{{\bar{x}}_{0}}\times 100$$

$${\bar{x}}_{t}=\mathrm{arithmetic}\;\mathrm{mean}\;\mathrm{of}\;\mathrm{the}\;\mathrm{two}\;\mathrm{replicate}\;\mathrm{gene}\;\mathrm{concentrations}\;\mathrm{at}\;\mathrm{the}\;\mathrm{respective}\;\mathrm{measurement}\;\mathrm{point}$$

Currently, no externally validated acceptance limits exist for the storage stability of iARG and exARGs in wastewater. Therefore, we derived target- and fraction-specific operational acceptance limits based on the variability observed in the baseline measurements (t0). This was done using the maximum allowable deviation (MAD) as outlined in ISO/TS 5667-25:2022 [40]:

(4) $$\mathrm{MAD}\left(\%\right)=\mathrm{MQD}\;\left(\%\right)=2{\mathrm{CV}}_{o}(\%)\sqrt{\frac{1}{r}+\frac{1}{q}}$$

*r* = *number of baseline measurements*

*q* = *number of replicates at each subsequent measurement point*

The resulting target- and fraction-specific acceptance interval was defined symmetrically around 100% as

*L* (%) = 100 − *MAD and U*(%) = 100 + *MAD* (5)

where (L) and (U) represent the lower and upper operational recovery limits, respectively. As the same baseline dataset was used for both temperatures, the corresponding target- and fraction-specific limits were applied to both 5 °C and −20 °C. Mean relative recovery rates within these limits were considered compatible with the study-specific analytical variability, whilst values outside the limits were classified as exceeding the operational stability criterion.

### 2.3. Data Analysis

Microsoft Excel 2019 MSO (Version 1808) and SigmaPlot (V15) were used to analyze the data, including descriptive statistics. All graphs were created with SigmaPlot (V15). The illustration (Figure 1) was created using Adobe Illustrator (30.0). The authors prepared the graphical abstract and visually refined it with assistance from ChatGPT (OpenAI, GPT-5.5 Thinking). No scientific data were generated, analyzed, or interpreted using GenAI.

## 3. Results

### 3.1. Method Evaluation

The basic physicochemical composition of the influent wastewater sample used for method comparison was as follows: ammonium nitrogen, 8.73 mg/L; total nitrogen, 19.3 mg/L; and chemical oxygen demand, 209 mg/L.

The total concentration of the different ARG targets (sum of GCs in iDNA-enriched (solid fraction) and exDNA-enriched (liquid fractions) per mL of wastewater) using the three sample preparation methods is shown in Supplement Table S8. Less common ARG targets, such as *bla*_OXA-48_ group, *bla*_VIM-1_ group, and *vanA,* are found at mean concentrations ranging from 2.3 × 10^2^ to 1.4 × 10^3^ GC/mL. More abundant targets, such as *sul1*, *QnrS1*, *intl 1*, and *tetW*, showed concentrations between 8.8 × 10^4^ and 1.9 × 10^6^ GC/mL. For filtration, centrifugation, and combined methods, at least 98% of the detected ARG concentrations were present in the iDNA-enriched fraction (Supplement Figure S3). Exceptions with lower proportions in the solid phase (iDNA) were *bla*_OXA-48_ group (54.1%), *tetW* (89.7%), and *QnrS1* (92.9%) using the centrifugation method.

**Figure 2:**
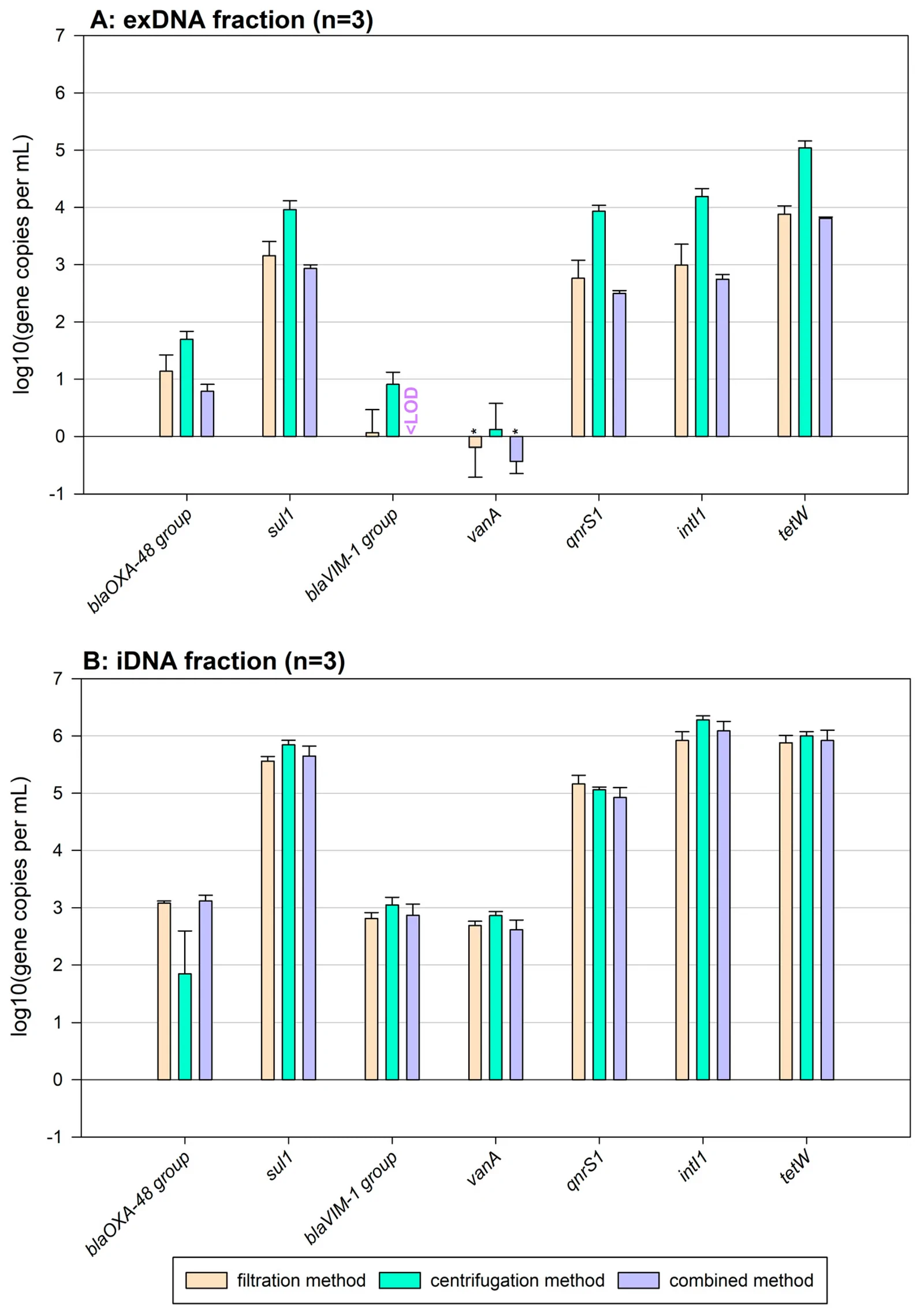
Target-specific gene concentrations [GC/mL] obtained using the three sample preparation methods. Concentrations are shown for (**A**) exDNA and (**B**) iDNA-enriched fractions. Bars represent the mean log10-transformed concentrations of three process replicates per method. The error bars indicate the corresponding SD on the log10 scale. <LOQ denotes values below the respective analytical limits, and <LOD denotes that dPCR detected no positive signal. (**A**) Filtration method: *vanA*: Only 1/3 of the replicates showed a quantifiable value. One replicate was below the LOD, whilst the other replicate was below the LOQ. (**A**) Combined method: *vanA*: Only 1/3 of the replicates showed a quantifiable value. 2/3 replicates showed a value <LOQ.

The target-specific presentation revealed different descriptive patterns for the iDNA- and exDNA-enriched fractions (Figure 2). In the exDNA-enriched fraction (A), the centrifugation method generally produced the highest mean log10-transformed concentrations across the quantifiable targets, whereas the filtration and combined methods yielded lower concentrations. This pattern was particularly apparent for *sul1*, *qnrS1*, *intI1*, and *tetW*. Concentrations of *bla*_VIM-1_ group and *vanA* in the exDNA-enriched fraction were close to or below the LOQ, and *bla*_VIM-1_ group was below the LOD when using the combined method.

In the iDNA-enriched fraction (B), the concentrations obtained with the three methods were generally more similar for *sul1*, *bla*_VIM-1_ group, *vanA*, *qnrS1*, *intI1*, and *tetW*. The most pronounced method-associated difference was observed for *bla*_OXA-48_ group, for which the centrifugation method yielded a lower mean concentration than the filtration and combined methods. Across the corresponding target–method combinations, iDNA concentrations were descriptively higher than exDNA concentrations.

Repeatability and descriptive total process recovery

Table 3 (Figure S2 in the Supplement) shows total process recovery rates for the total target concentration using *E. coli* as the target.

**Figure 3:**
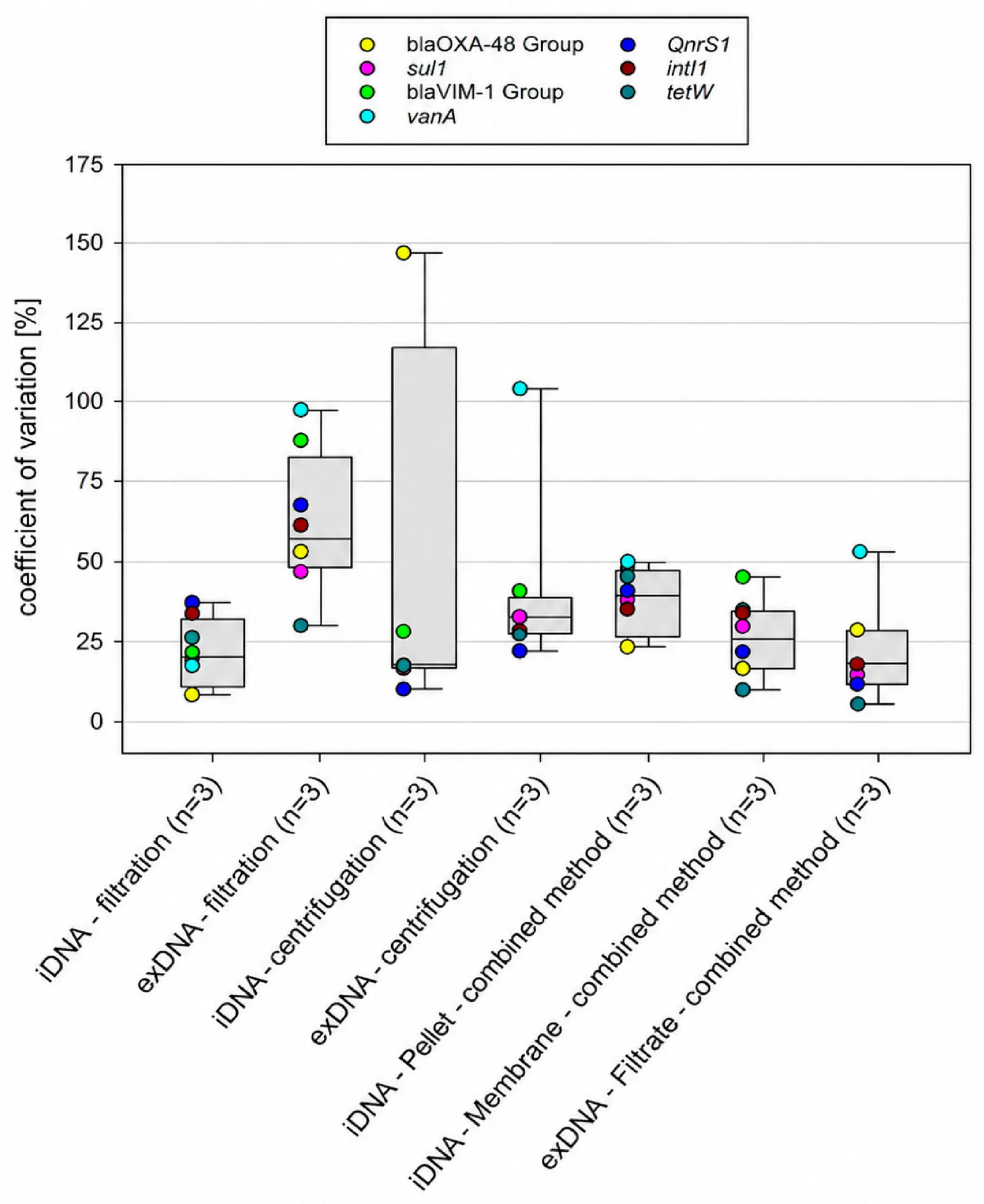
Jittered box plots of coefficients of variation [%] of ARG targets (*bla*_OXA-48_ group, *sul1*, *bla*_VIM-1_ group, *vanA*, *QnrS1*, *intl1*, and *tetW*) between process replicates (n = 3) of the different fractions (iDNA and exDNA) of filtration, centrifugation, and the combined method. The median is shown in the box, and 10th and 90th percentiles are shown as whiskers.

Repeatability, determined from three process replicates, is shown as CVs in Figure 3. CVs ranged from 5.5% to 146.9%; for most targets, CVs were below 50%. Some targets and fractions showed higher CVs. This was particularly true for both fractions of the centrifugation method: the *vanA* and *bla*_OXA-48_ group showed CVs higher than 100%. Furthermore, the precision of the exDNA-enriched fraction in the filtration-based method was lower compared to the other two exDNA-enriched fractions.

Cost comparison

The 2024 local cost estimate for consumables used to extract iDNA and exDNA from a process replicate was compared for filtration, centrifugation, and combined methods, excluding downstream dPCR analysis. The cost of dPCR analysis of an extract using the materials employed in our study is approximately 8.50 [€] (based on prices in 2024), based on the following assumptions: SinglePlex assay (one target); no technical replicates; commercial assay; no positive or negative controls; and analysis using QIAcuity Nanoplate 26,000 (QIAcuity Nanoplate 26k 24-Well, QIAGEN GmbH, Hilden, Germany). The cost of dPCR analysis increases with the number of targets analyzed.

The main cost drivers were the commercial extraction kits used for iDNA and exDNA extraction. In particular, exDNA extraction contributed substantially to the overall costs, as it was performed using a partly automated commercial extraction workflow. In contrast, differences between the sample preparation methods were mainly related to method-specific consumables and handling steps, such as filtration materials or centrifugation supplies.

Table 4 summarizes the cost comparison of these sample preparation approaches. Filtration and centrifugation methods were comparable in cost per sample. The combined method was more resource-intensive due to the additional separation step (pellet and membrane for the iDNA-enriched fraction), and costs were 15% higher than with filtration or centrifugation alone. The proportion of costs for exDNA extraction relative to the total costs for exDNA and iDNA extraction is 85% for filtration and centrifugation and 77% for the combined method.

Usability, Operational time, Qualitative workflow, and resource observations

Table 5 and Table 6 summarize the operational time, qualitative workflow, and resource observations of filtration, centrifugation, and the combined method. The centrifugation method achieved an SUS of 60, whereas both the filtration and combined methods achieved higher scores of 72.5; a higher score indicates a more favorable perceived workflow handling. For more details, see Table S2 in the Supplementary Material.

The time-in-motion metrics (Table 5) showed very similar values across all three methods, indicating that hands-on time and total turnaround time did not differ substantially between the extraction approaches. Run-associated cleaning time differed among the three methods, with the centrifugation method requiring approximately 2.5 h less time than the other two methods. Filtration and combined methods required substantially longer run-associated cleaning and decontamination time (4 h each) because they included autoclaving and sterilization of the filter cartridge in addition to the decontamination program for the automatic extraction unit. In contrast, the centrifugation method required only 1.5 h, as cleaning and decontamination were limited to the automatic extraction unit’s decontamination program.

Table 6, also showed largely comparable qualitative workflow and resource observations, including required equipment inventory and resource metrics. However, differences became more apparent when examining run-associated cleaning, workflow-specific handling parameters, and the usability score (SUS).

### 3.2. Storage Stability Test

Storage stability over 63 days at two temperatures (5 °C and −20 °C) was tested using a second wastewater sample. To define the concentration immediately after sampling and before storage (time point t0), 11 process replicates were processed with the combined method. Using this method, 11 iDNA extracts and 10 exDNA extracts were prepared and further analyzed by dPCR. Due to an error in sample handling during exDNA extraction, one replicate was missing compared with iDNA.

Characterization for time point t0 and the corresponding descriptive statistics are shown in Figure S4 and Table S3 in the Supplementary Materials and reveal concentrations comparable to those observed in the method comparison. When examining the target *bla*_VIM-1_ group in the exDNA-enriched fraction, eight process replicates showed concentrations below the LoQ but positive partitions. For the ARG *vanA*, six of ten process replicates showed a concentration below the LoQ but positive partitions, and two of ten process replicates showed a concentration below the LoQ and no positive partitions. These two process replicates could not be included in any further analysis of the ARG *vanA*. The CVs [%] range from 10% (*tetW*) to 29% (*vanA*) in the iDNA-enriched fraction and from 26% (*tetW*) to 56% (*bla*_OXA-48_ group) in the exDNA-enriched fraction (Figure S4 and Table S3).

**Figure 4:**
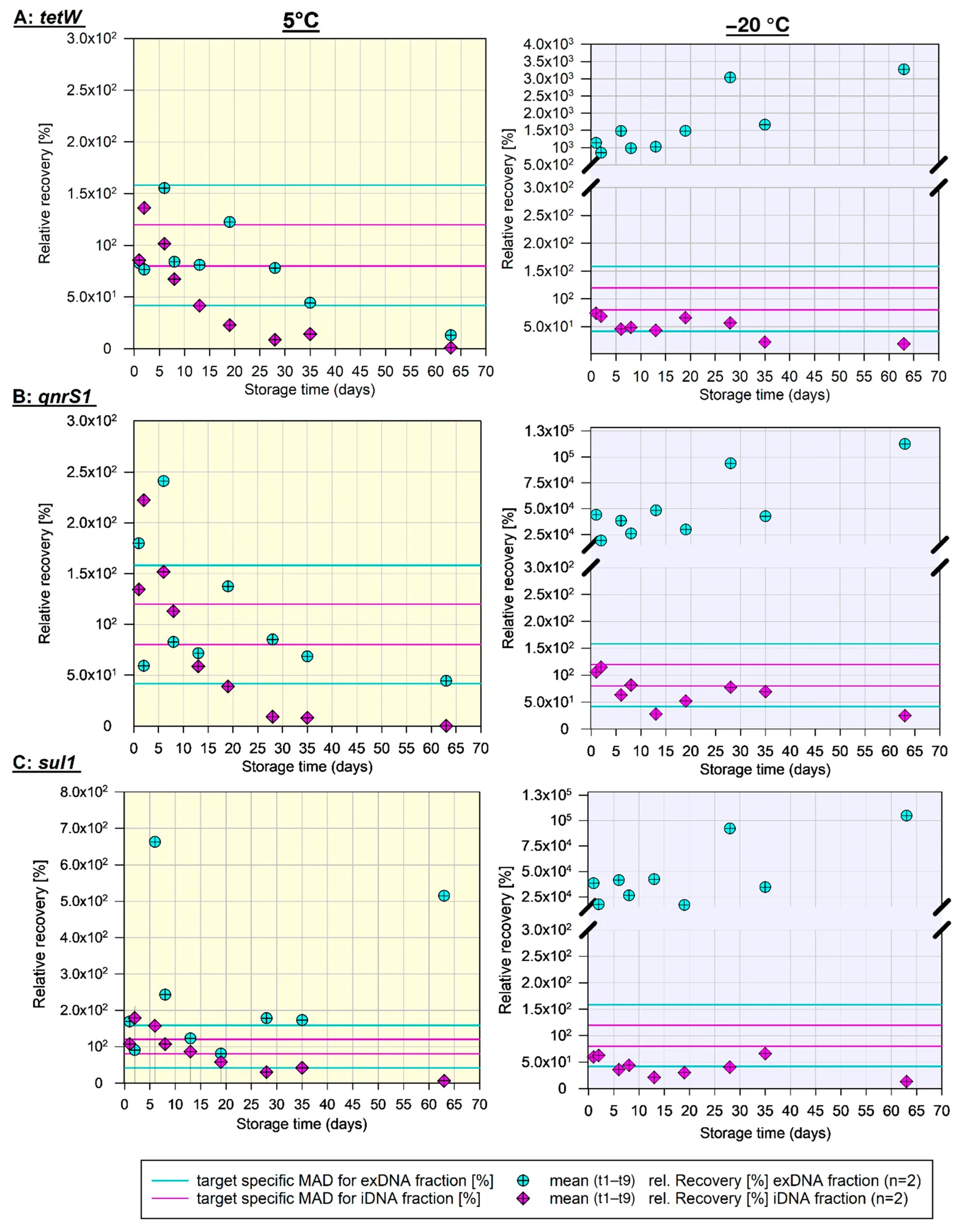
Relative recovery of ARG concentrations in the iDNA-enriched and exDNA-enriched fractions during storage at 5 °C and −20 °C. The rows represent the ARG target genes *sul1*, *tetW*, and *qnrS1*, whilst the columns represent the two storage temperatures (yellow = 5 °C, purple = −20 °C). The relative recovery [%] was calculated for each observation as the ratio of the concentration at the storage time point t to the corresponding target- and fraction-specific mean of the concentration at t0. The common t0 reference comprised replicates for iDNA (n = 11) and exDNA (n = 10); t1–t9 each comprised two replicates per fraction and storage temperature. The purple (iDNA) and blue (exDNA) horizontal lines indicate the target- and fraction-specific limits for the maximum allowable deviation (MAD).

Based on the results at time point t0 (Figure S4), we calculated the mean and the CVs for target- and fraction-specific limits for the maximum allowable deviation (MAD) (Table S9) as reference limits for the results of subsequent time points (t1–t9; n = 2). Results of the subsequent time points (t1–t9) are shown in Figure 4 and Supplement Figure S6.

For all three targets shown in Figure 4, a similar temporal pattern was observed at 5 °C. The mean relative recovery of the iDNA-enriched concentration initially increased or remained close to 100% before decreasing during continued storage. However, the time at which the mean recovery fell below the target-specific lower MAD limit differed among the targets. The mean iDNA-enriched recovery of *tetW* fell below the lower MAD limit on day 8 (t4), followed by *qnrS1* on day 13 (t5) and *sul1* on day 19 (t6). Once below the lower MAD limit, the mean iDNA-enriched recoveries remained below their respective limits at all subsequent measurement points. By day 63 (t9), the mean iDNA-enriched recoveries of all three targets had decreased to approximately 0–5%.

The decrease was less consistent in the exDNA-enriched fraction during storage at 5 °C. The mean exDNA-enriched recovery of *tetW* remained above its lower MAD limit until day 35 (t8) but fell below the limit on day 63 (t9). For *qnrS1*, the mean exDNA-enriched recovery on day 63 was close to the lower MAD limit, with the two individual observations located on opposite sides of the limit. The mean exDNA-enriched recovery of *sul1* did not fall below its lower MAD limit during the investigated storage period. However, several exDNA values, particularly for *qnrS1* and *sul1*, exceeded their respective upper MAD limits. Thus, the exDNA-enriched results at 5 °C showed substantial variation rather than a uniform decrease.

Storage at −20 °C resulted in a clear divergence between the relative recoveries of the two operational DNA fractions. For *tetW*, the mean iDNA-enriched recovery was below its lower MAD limit at every time point (t1–t9). For *qnrS1* and *sul1*, the mean iDNA-enriched recovery fell below the respective lower MAD limits from day 6 (t3) onwards and remained below these limits through day 63 (t9). At t9, mean iDNA-enriched recoveries were approximately 20% for *tetW*, 25% for *qnrS1*, and 15% for *sul1*.

In contrast, mean exDNA-enriched recoveries at −20 °C exceeded their respective upper MAD limits from day 1 (t1) onward and remained above these limits throughout the storage period. Maximum mean relative recoveries reached approximately (3.4 × 10^3^%) for *tetW*, (1.15 × 10^5^%) for *qnrS1*, and (1.05 × 10^5^%) for *sul1*.

## 4. Discussion

Monitoring of antimicrobial resistance (AMR) in wastewater is increasingly important. This is also evidenced by the requirements of the Urban Wastewater Treatment Directive (Directive (EU) 2024/3019). However, no standards currently exist for monitoring AMR in wastewater, and it is not yet defined whether influent and/or effluent samples from WTTPs should be included in AMR monitoring, or whether exDNA should be analyzed alongside iDNA [11,24]. To assess high-risk ARGs and evaluate AMR emissions from WWTPs, a joint analysis of iDNA and exDNA is recommended [16,41]. Thus, workflows that detect ARGs and differentiate between exDNA and iDNA fractions are vital for developing appropriate monitoring instruments for AMR in wastewater and other environmental samples.

In this study, we presented data from an exploratory comparison of three different preparation methods for the parallel analysis of iDNA- and exDNA-enriched DNA fractions. Our study provides data on operational strengths and limitations of the different methods, offering insights and practical information for the implementation of workflows for the parallel analysis of iDNA and exDNA in wastewater samples. Additionally, we provide information on the storage stability of the different DNA fractions and ARGs and present preliminary data indicating that storing municipal wastewater samples at 5 °C for up to six days is acceptable for most of the targets examined.

### 4.1. Method Evaluation

Repeatability and total process recovery

Repeatability of the method measured as CVs between process replicates showed high variability in our study: we found CVs for the iDNA-enriched fraction in the range from 8.5–37.2% ((1) filtration method), 10.2–146.9% ((2) centrifugation method), and 9.9–49.9% ((3) combined method) and for the exDNA-enriched fraction, respectively: (1) 30.0–97.5%, (2) 22.1–104.3%, and (3) 5.5–53.1%. At low exDNA concentrations, however, the calculated CVs were strongly influenced by censoring and the substitution of values below the LOQ with half the LOQ and should therefore be interpreted with caution. For the centrifugation method, a particularly high CV was observed for the ARG *bla*_OXA-48_ group in the iDNA-enriched fraction (146.9%); this value is likely an outlier, given that one out of three process replicates displayed implausibly high concentrations (493 GC/mL).

Direct literature benchmarks for CVs (%) between process replicates in ARG analyses of wastewater are limited, as most studies report variability between technical replicates or inter-laboratory variability, rather than CV values for process replicates. Published qPCR data [42] indicate variability of 3–28% between technical replicates under inter-laboratory conditions. Liguori et al. [43] report substantial variation among process replicate samples for the targets *sul1* and *intI1* in the iDNA-enriched fraction, in some cases exceeding 50% CVs. Our results are comparable, indicating acceptable precision values under the given conditions [43].

Process recovery of the total concentration of *E. coli* (GC/mL) ranged from 38% to 43.5% (median) across the three methods, indicating moderate recovery across the complete workflows.

Chen et al. [44] reported comparable recovery rates of 28% to 42% from the iDNA fraction in biosolid samples using the same commercial extraction kit as in our study. Zöhrer et al. [45] investigated complex solid samples (soil, sediment, sludge, compost) and applied a comparable spike-and-recovery approach using *E. coli* as spike material and a commercial extraction kit combined with dPCR detection. They reported much lower recoveries (mean 4.9% ± 4.34%; exDNA 4.8% ± 6.78%; iDNA 7.8% ± 7.01%) [45]. Fretschner et al. [46], investigating the recovery of viral targets in wastewater influents and using comparable workflows with the same commercial extraction kits, reported recoveries of 44.4%, 37.1%, and 37.9% at high, medium, and low spike levels, respectively, which aligns with our results. Overall, the recovery range of 38–42% appears plausible for a complex wastewater matrix. However, recovery rates reported in the literature for exDNA extraction vary widely, ranging from <10% to 90% [45,47,48,49,50].

Three limitations constrain the interpretation of the recovery results. First, the limited and unequal numbers of observations (filtration, n = 3; centrifugation, n = 3; combined method, n = 2) do not support formal statistical comparisons or conclusions regarding equivalent method performance. Second, recovery was determined for the complete workflow as the sum of the iDNA- and exDNA-enriched fractions; therefore, it does not provide fraction-specific recovery estimates. Third, an *E. coli* spike rather than an ARG target was used, precluding conclusions about target-specific ARG recovery. Accordingly, the recovery results should be considered descriptive and preliminary.

To date, no easy-to-use, reliable process controls for PCR-based monitoring of ARGs in wastewater are available. Thus, because of its ease of handling in the laboratory and its ubiquitous presence in wastewater, *E. coli* seems to be a useful alternative for process control [51]. Although phage-like particles have been proposed and evaluated as process controls for RT-qPCR-based detection of enteric RNA viruses, analogous standardized process controls specifically designed for PCR-based ARG monitoring in wastewater are still lacking [52,53].

Cost comparison

Direct cost benchmarking for sample pretreatment and the extraction of iDNA and exDNA against the literature data was not feasible, as existing studies primarily report analytical performance metrics rather than comprehensive, standardized per-sample cost data. Although methods exist to separate iDNA and exDNA from a single water sample simultaneously, the lack of cost reporting hinders a sound economic assessment of the various approaches. This represents a relevant gap in the context of the upcoming implementation of the revised Urban Wastewater Treatment Directive, where standardized monitoring of antimicrobial resistance will require not only harmonized analytical methods but also a clearer picture of the associated costs.

Because the filtration and centrifugation methods for simultaneous iDNA and exDNA extraction were less complex than the combined method, they were more cost-efficient. It also became apparent that most total costs were incurred during exDNA extraction, accounting for 77–85% of workflow costs. The high costs associated with exDNA extraction in our study were mainly due to the use of a commercial kit in combination with automated extraction. In contrast, when manual precipitation methods, such as ethanol precipitation with sodium acetate followed by extraction with a commercial kit, were applied, as described by Ficetola et al. [37], the costs were considerably lower, amounting to approximately €20 for the combined extraction of iDNA and exDNA per process replicate, excluding dPCR analysis. However, a method comparison in the context of wastewater monitoring for influenza viruses by Fretschner et al. [46] showed that commercial filtration-based kits combined with automated extraction achieved substantially higher recovery rates than workflows combining precipitation with commercial automated extraction. Thus, while precipitation-based approaches may reduce costs, their potential impact on recovery and workflow robustness has to be considered.

The costs associated with iDNA extraction were comparable to those reported in the literature. For contextualization, Pham et al. [54] reported costs of $4.48 per sample of environmental freshwater for filtration and iDNA extraction using a commercial kit.

Usability, Operational time, Qualitative workflow and resource observations

In addition to costs, we assessed practical handling to evaluate the suitability of the three workflows for routine application. Usability of the extraction workflows was evaluated using the System Usability Scale (SUS) [55,56,57,58]. The SUS is a widely used, technology-agnostic questionnaire originally developed to provide a quick global assessment of perceived usability. It has primarily been applied in fields such as human–computer interaction and user experience research, including consumer and enterprise software, websites, mobile phones, and other technical systems [55,56,57,58]. We used the SUS in our present study and adapted it to assess the perceived usability and practical workflow handling of laboratory extraction methods. Consequently, the SUS results should be interpreted as a subjective measure of workflow usability. However, this instrument may be valuable for evaluating the handling of analytical methods. The commonly cited SUS average of approximately 68 is derived from broad usability benchmark datasets across heterogeneous technical systems, including software, websites, mobile devices, hardware, and other user-facing systems. Thus, in the present study, this value should only be used as a general reference point for perceived usability. Results indicate a more favorable perceived workflow handling for the filtration-based and combined methods compared with the centrifugation method. The filtration and combined methods showed slightly higher perceived usability than the commonly cited SUS average [55,56,57,58]. A limitation of our study is that only one independent operator took part in the SUS survey. Future studies should increase the number of respondents.

The qualitative workflow parameter provided additional context for these usability differences. The centrifugation method showed higher perceived risks of pipetting complexity, cross-contamination between liquid and solid phases, and sample loss during processing compared with the filtration and combined methods. These factors likely reflect the additional handling steps required for pellet processing and liquid transfers during centrifugation-based concentration procedures. In contrast, the filtration method was associated with a higher risk of filter clogging, a common operational challenge in filtration-based sample concentration that may negatively affect workflow reliability in routine laboratory practice.

### 4.2. Storage Stability Test

Application of the two-sided, target- and fraction-specific MAD limits showed that the duration for which relative recovery remained within the operational acceptance range differed among targets, DNA fractions, and storage temperatures. No common acceptable holding time was identified across all targets and DNA fractions when both the lower and upper MAD limits were considered. Nevertheless, the study-specific operational acceptance criteria allowed derivation of provisional target-, fraction-, and temperature-specific holding times. When only decreases in relative recovery were considered, storage for up to six days at 5 °C did not result in a mean recovery below the corresponding lower MAD limit for any target or DNA fraction. Accordingly, conclusions regarding storage stability were regarded as preliminary and specific to the target substances under investigation.

To the best of our knowledge, only a few studies have explicitly investigated the stability of ARGs in the exDNA fraction as a function of storage temperature and duration in raw wastewater samples [17,25,59]. Our results align with recommendations from established molecular monitoring protocols. According to U.S. EPA Method 1609, water samples intended for PCR analysis should be kept on ice or refrigerated below 10 °C during transport and should not be frozen before analysis [60]. Similarly, the 2025 WHO pilot guidance identifies conventional cold-chain transport as the default for AMR wastewater surveillance, while noting that transport and storage conditions may introduce method-dependent biases and should be evaluated for the intended analytical approach [32]. Keenum et al. [23] and Schwermer et al. [31] similarly state that freezing before the concentration step is not recommended, as it may damage cells and reduce the effectiveness of subsequent DNA capture by filtration, although this explanatory wording is not stated verbatim in the EPA method itself. This is consistent with other studies on the stability of DNA for PCR analysis, which also recommend storing water samples at 4 °C. In line with this concern, Gilpin et al. [61] reported that freezing a water sample at −20 °C resulted in an immediate 1-log loss of PCR signal from spiked DNA, although the remaining signal was stable for at least three months afterward. Gilpin et al. [61] also showed that storing the water sample at 4 °C led to an initial 0.5-log reduction in the PCR product in the iDNA fraction within the first week, followed by a further exponential decrease during the experiment. A similar trend was observed in the present study for the targets *tetW* and *QnrS1* in the iDNA-enriched fraction. Hinlo et al. [62] further demonstrated that frozen water samples showed significantly fewer DNA PCR signals in the iDNA fraction than samples stored at 4 °C from day 2 onward, corresponding to one freeze cycle. Also, studies on SARS-CoV-2 RNA stability in wastewater suggest that refrigerated storage at 4 °C is a cautious and practical option when samples are analyzed within a few days [63].

Our results show that the exDNA-enriched fraction seems to be more stable than the iDNA-enriched fraction. iDNA in aquatic environments or wastewater may initially be protected within intact bacterial cells. Upon cell lysis, released DNA enters the exDNA pool, where its persistence depends strongly on environmental conditions and the degree of structural protection. In particular, particle-associated or otherwise protected exDNA may be substantially more stable than freely dissolved exDNA in aquatic environments [5,64,65]. The previous literature generally recommends that water samples intended for PCR analysis should be processed, or at least filtered, within 24 h [31,61].

Studies by Zhang et al. [17] and Wang et al. [25], which investigated the persistence of ARGs in exDNA in wastewater at 20 °C and 21 °C, respectively, showed that cell-free extracellular ARGs can persist in wastewater for extended periods, remaining detectable over prolonged storage and exhibiting only limited degradation even after 25 days. Although these studies were conducted under different experimental conditions and did not directly correspond to the refrigerated storage conditions applied in the present study, they indicate that exDNA may contribute to the long-term persistence and potential environmental dissemination of ARGs in wastewater-associated environments [17,25].

Calero-Cácerese et al. [59] demonstrated the stabilizing effect of refrigerated storage on *sul1* in raw wastewater samples, but also indicated fraction-specific differences. They quantified *sul1* in both the bacterial and bacteriophage fractions of raw wastewater and reported only minor deviations during storage at 4 °C for up to 43 days [59]. In contrast, concentration reductions were observed during storage at 22 °C and 37 °C, and ARG persistence was generally higher in the bacteriophage fraction than in the bacterial fraction. In the present study, *sul1* concentrations in the exDNA-enriched fraction remained largely unchanged during storage at 5 °C for up to 63 days, consistent with the high stability reported under refrigerated conditions. In contrast, *sul1* concentrations in the iDNA-enriched fraction declined from day 19 (t6) onwards, suggesting that the cell-associated fraction in our experimental setup was more sensitive to prolonged storage than the extracellular fraction. However, comparability is limited, as the bacteriophage fraction analyzed by Calero-Cácerese et al. [59] represents capsid-protected extracellular DNA and is therefore not directly equivalent to the exDNA-enriched fraction investigated in the present study. Nevertheless, both studies consistently suggest that non-cell-associated ARG pools, particularly when structurally protected, may be at least as stable as, and potentially more stable than, cell-associated DNA under refrigerated conditions [59]. To evaluate the results of our storage experiment, we applied ISO/TS 5667-25:2022. However, ISO/TS 5667-25:2022 formally excludes biological and microbiological methods from its scope [40]. Consequently, the standard’s framework was applied only by analogy to evaluate the storage stability of the investigated DNA targets and does not constitute a standard-compliant validation. The resulting MAD limits should therefore be regarded as study-specific operational acceptance criteria rather than generally applicable limits established in accordance with the standard [40]. Other approaches to stability assessment have also been described. For example, Tavazzi et al. used an isochronous design and linear regression to evaluate SARS-CoV-2 RNA targets in wastewater, with instability indicated by a significant slope and shelf life estimated using a predefined uncertainty threshold of 20% [63]. Although not directly transferable to the ARGs investigated here, this study illustrates an alternative regression-based approach to storage-stability assessment.

### 4.3. Methodological Challenges and Limitations

The following limitations and methodological challenges should be considered when interpreting the results of the present study.

The WHO emphasizes that differences in sampling, transport, storage, extraction, and analytical workflows can substantially affect the detected AMR and ARG profiles and should therefore be standardized or explicitly accounted for [32]. It also recommends context-specific assay validation with adequate sensitivity and specificity. Accordingly, this comparison represents an initial step toward workflow selection and optimization rather than a full validation of the three methods. The method comparison was conducted as a single-matrix pilot study and was not designed as a formal validation study. Wastewater composition, suspended solids, turbidity, extracellular DNA content, and the abundance of PCR-inhibitory substances can vary substantially among sampling locations and over time. Consequently, the observed results are specific to the wastewater matrix and experimental conditions investigated.

A further limitation was the standardized 80 mL sample volume, which reflected the processing capacity of the exDNA extraction kit and was maintained across experiments to ensure comparability. Each extract was analyzed in a single dPCR reaction and at one additional dilution, which was not considered an independent technical replicate. Consequently, we could not assess variability arising from reaction preparation and pipetting separately.

Our study relied solely on targeted PCR methods. Consequently, only predefined ARG targets were quantified, whereas broader resistome-level information and emerging AMR determinants were not captured. Future studies could therefore complement targeted PCR approaches with metagenomic analyses to obtain a more comprehensive overview of AMR determinants in wastewater. A position paper published in *Eurosurveillance* describes culture-based analyses, targeted PCR, metagenomic sequencing, and liquid chromatography–mass spectrometry as complementary approaches whose relevance depends on the intended surveillance objective [24].

Nevertheless, PCR-based methods remain useful for sensitive, quantitative surveillance of predefined priority ARGs when applied in line with clearly defined monitoring objectives. Hock et al. [24] emphasized that target selection should be guided by the intended purpose of surveillance, including assessing population-level AMR trends using WWTP influent, AMR emissions using WWTP effluent, and AMR reduction efficiency across wastewater treatment processes. Similarly, the 2025 WHO pilot guidance states that targeted qPCR or dPCR can improve the sensitivity of detecting priority ARGs at low concentrations [32]. However, the WHO also emphasizes that detection of an ARG alone does not establish its human origin, association with a viable and clinically relevant host, expression, or contribution to phenotypic resistance. ARG detection should therefore be interpreted as target-specific molecular surveillance rather than as directly actionable public health evidence. Translation into public health action would require validated methods, predefined surveillance objectives and response triggers, and integration with relevant phenotypic, clinical, and broader One Health surveillance data [32].We measured high average total concentrations (iDNA + exDNA) of the integron integrase gene *intI1* and the ARGs *sul1* and *tetW,* which is consistent with their use as indicators of anthropogenic inputs and their ubiquitous presence in wastewater [23,28,29]. In contrast, the lower total concentrations of *bla*_VIM-1_ group, *bla*_OXA-48_ group, and *vanA* may reflect their association with less commonly used “last-resort” antibiotics [20,23]. In our study, all targets were detected with all three methods, regardless of their frequency. ARG concentrations in the iDNA- and exDNA-enriched fractions were broadly comparable across the three investigated sample pretreatment methods and largely consistent with those reported in previous wastewater studies. For several targets, including *qnrS1* and *sul1*, concentrations in the exDNA and iDNA-enriched fractions were within the literature ranges for raw municipal wastewater [8,14,41,47,66,67]. In contrast, *intI1* and *bla*_VIM_ tended to occur at lower concentrations in the exDNA-enriched fraction than reported in other studies investigating municipal and clinical wastewater [20,41,66,67]. For *bla*_OXA-48_ group, the concentrations measured in this study (5 × 10^0^–4 × 10^1^ GC/mL) were lower than those reported by Oliveira et al. [41] for Portuguese WWTP influents with a range from 5.6 to 9.0 x 10^2^ GC/mL, but comparable to those observed by Erler et al. [20] in hospital wastewater in Germany (3 × 10^0^–3 × 10^2^ GC/mL). In this method comparison, we examined only two 24 h wastewater samples (one in summer and one in autumn), which provides only a snapshot of ARG occurrence in municipal wastewater. Differences in measured concentrations can also be explained by differences in catchment area and geographical location, leading to different antibiotic-consumption patterns [33] and environmental conditions such as temperature [68].

Furthermore, differences may be attributed to methodological differences [68]: In contrast to Savin et al. [8], Erler et al. [20], and Oliveira et al. [41], we used a direct-capture method to extract exDNA. Furthermore, we used dPCR rather than qPCR. The broad range of exDNA concentrations and extraction methods across studies highlights the need for standardized, workflow-compatible protocols to improve data quality and comparability [8,12,14,16,17,18,20,41,69].

In the present study, at least 90% of the total ARG concentration (iDNA + exDNA) was detected in the iDNA-enriched fraction. This is consistent with results from several other studies reporting a stronger representation of individual ARGs in the iDNA fraction and generally low ARG concentrations in the exDNA fraction of raw wastewater [8,14,17,20,41,47,66]. However, Zhang et al. [17] reported persistence of exDNA during wastewater treatment and an increasing exDNA-to-iDNA abundance ratio across the treatment process, indicating a potential enrichment of ARGs associated with free exDNA.

In the present study, we used filter- and centrifuge-based approaches to distinguish between cell-associated and extracellular DNA fractions. This separation should be interpreted as operational rather than absolute, as both approaches separate DNA fractions based on particle size, cell association, and sedimentation behaviour. Consequently, overlap between the fractions cannot be completely ruled out. Particle-bound exDNA or extracellular DNA adsorbed onto suspended matter may be retained on filters or sediment with the pellet, and thus be assigned to the iDNA fraction [5,14]. Conversely, incomplete removal of cells or fine particles may influence the composition of the exDNA fraction. In our study, we cannot rule out misclassifications between iDNA and exDNA. Additional steps, such as DNase treatment of the iDNA-enriched fraction, could help characterize the purity of the separated fractions more effectively, as this could separate free, particle-bound exDNA, for example, on the filter or the pellet, from the iDNA [5,14].

Finally, sample storage represents an additional methodological factor that can influence the interpretation of iDNA and exDNA data. Although the available literature is not fully consistent and direct comparisons remain limited, the evidence suggests that iDNA may initially be protected within intact cells in aquatic environments. Upon cell lysis, released DNA enters the exDNA pool, where its persistence depends strongly on environmental conditions and the degree of structural protection. In particular, particle-associated or otherwise protected exDNA may be substantially more stable than freely dissolved exDNA in aquatic environments [5,64,65]. The previous literature generally recommends that water samples intended for PCR analysis should be processed, or at least filtered, within 24 h [31,61]. If immediate processing is not possible, refrigerated storage for approximately 3–5 days has been recommended [62].

## 5. Conclusions

We evaluated three different methods for preparing wastewater samples for the combined detection of intracellular and extracellular ARGs. Using relevant ARGs with different abundances, we compared a filtration-based method, a centrifugation-based method and a combined method. In our study, all sample preparation workflows were suitable for simultaneous detection of iDNA and exDNA. The proportion of iDNA-enriched fraction in total DNA was approximately 90% or higher across all methods and for most target sequences. The repeatability (CVs) for the filtration-based method and the combined method appears to be better than those for the centrifugation-based method. However, CVs for the iDNA- and exDNA-enriched fractions varied widely between process replicates, again highlighting the importance of sample homogenization and preparation [43,70].

Furthermore, we investigated costs, usability, operational time, and qualitative workflow and resource observations for the different methods. Regarding usability, qualitative workflow, and resource observations, the filtration-based and combined methods were slightly superior. The centrifugation method was associated with higher perceived risks, including pipetting complexity, cross-contamination between liquid and solid phases, and sample loss during processing. Consumable costs were comparable for the filtration and centrifugation methods but about 15% higher for the combined method. Although the combined method was somewhat more cost-intensive, the better repeatability, improved qualitative workflow observation, and usability appear to outweigh this disadvantage.

Evaluating the stability of a wastewater sample under different storage conditions and durations was another issue in our investigations. Moreover, the results suggest that refrigerated storage of wastewater samples for up to six days may be practical for PCR-based analysis of ARGs in the iDNA- and exDNA-enriched fractions. The results also suggest that ARGs in the exDNA-enriched fraction may be more stable, with detectability maintained for up to 35 days of storage. Storage at −20 °C, however, is not recommended due to the demonstrated misclassification of the iDNA- and exDNA-enriched fractions.

## Figures and Tables

**Table 1:** Operational definitions of the domains used to characterize the three sample preparation methods.

Evaluation Domain	Explanation
Repeatability	Repeatability was defined as the variation among replicate aliquots processed under the same conditions, including the same operator, laboratory, equipment, analytical run, and extraction protocol. It was calculated separately for each method, DNA fraction, and target as the coefficient of variation (CV) [%]. For the combined method, the membrane-derived and pellet-derived iDNA-enriched fractions were analyzed separately to allow direct comparison with the corresponding iDNA-enriched fractions obtained by filtration and centrifugation.
Descriptive total-process recovery	Recovery was assessed using wastewater samples spiked with a known concentration of *Escherichia coli*. It was calculated for the complete processing and extraction workflow as the sum of the concentrations measured in the iDNA-enriched and exDNA-enriched fractions and expressed as recovery (%) = 100 × recovered total concentration/spiked concentration. Recovery was based on n = 3, n = 3, and n = 2 observations for the three methods, respectively.
Cost	Local 2024 consumables cost estimate for extracting iDNA and exDNA from one process replicate. It excludes labor, equipment amortization, energy, waste disposal, quality controls, technical repetition, and complete dPCR costs.
Usability	Operator-perceived usability was assessed using the System Usability Scale (SUS), which provides a score ranging from 0 to 100, with higher scores indicating a more favorable perceived workflow. One operator completed the SUS questionnaire after performing each extraction workflow. Because only one operator participated, SUS results were treated as descriptive, method-oriented exploratory observations and were not subjected to inferential statistical analysis.
Operational time	Operational time was assessed by time-in-motion recording and included hands-on time, total turnaround time, and separately recorded run-associated cleaning time. Turnaround time comprised the entire period from the start of sample processing until the iDNA and exDNA eluates were ready for downstream analysis.
Qualitative workflow and resource observations	Qualitative workflow and resource observations comprised characteristics that were not converted into a numerical score. These included workflow complexity, filtration or centrifugation requirements, susceptibility to workflow interruptions such as membrane clogging, ease of sample transfer, cleaning requirements, required equipment, operator qualification, and laboratory infrastructure. These observations were recorded during implementation of each workflow and were interpreted descriptively rather than as measures of repeatability, recovery, usability, or cost.

**Table 2:** Description of aliquots and actual storage time in days.

Designation Measurement Point	Actual Storage Time [Days]	Number of Process Replicates for 5 °C	Number of Process Replicates for −20 °C
T1	1	2	2
T2	2	2	2
T3	6	2	2
T4	8	2	2
T5	13	2	2
T6	19	2	2
T7	28	2	2
T8	35	2	2
T9	63	2	2

**Table 3:** Recovery rates for the filtration, centrifugation, and combined method for the total concentration using *E. coli* as the target. Individual recovery rates and means with corresponding 95% confidence intervals (CIs) are presented for each method.

Method	Recovery 1 [%]	Recovery 2 [%]	Recovery 3 [%]	Median [%]	Mean [%]	95% CI
Filtration Method (n = 3)	43.5	38.6	62.2	43.5	48.1	[17.2–79.0]
Centrifugation Method (n = 3)	38.4	26.4	40.4	38.4	35.0	[16.3–53.8]
Combined Method (n = 2)	39.7	45.4	-	42.6	42.6	[6.1–79.0]

Data are presented as individual recovery rates and as means with 95% confidence intervals calculated using Student’s t-distribution (n = 3 resp. n = 2).

**Table 4:** Estimate of local consumable costs for 2024.

	Method	Cost for Extraction iDNA from Solid Phase (Filter; Pellet) per Process Replicate Without dPCR Analysis [€]	Cost for Combined Extraction of iDNA and exDNA per Process Replicate Without dPCR Analysis [€]
1	filtration; analysis of filter and filtrate	8.07	52.85
2	centrifugation; analysis pellet and supernatant	7.22	52.84
3	Combined method; analysis pellet, membrane, and supernatant.	14.46	60.47

Consumables cost estimate only. This estimate excludes labor, equipment amortization, energy, waste disposal, quality controls, technical repetition, and complete dPCR costs. A detailed overview of the costs for each method per process replicate can be found in the Supplementary Tables S4–S6.

**Table 5:** Summary of operational time.

Time-in-Motion	Filtration	Centrifugation	Combined Method
Hands-on time (HoT)	3:15 h by two operators	2:45 h by two operators	3:00 h by two operators
Total turnaround time (TaT)	6 h by two operators	5 h by two operators	6 h by two operators
Run-associated cleaning time	4 h	1.5 h	4 h

**Table 6:** Summary of qualitative workflow and resource observations for the method comparison of the filtration, centrifugation and combined method.

	Filtration	Centrifugation	Combined Method
Number of process replicates analyzed:	6 (n = 3 spiked; n = 3 unspiked)	6 (n = 3 spiked; n = 3 unspiked)	6 (n = 3 spiked; n = 3 unspiked)
**Overhead**			
Required equipment inventory	No major differences in the number of devices required.		
Resource required qualification	Routine laboratory staff/scientists or laboratory staff after training		
Infrastructure	No major differences.		
**Workflow** **parameter**			
Number of operators	2	2	2
Pipetting complexity	Moderate	High	Moderate
Risk of cross-contamination between liquid and solid phase	Low	High	Low
Risk of sample loss during processing	Moderate	High	Moderate
Risk of filter clogging	High	n/a	Low
Pellet handling required	n/a	Yes	Yes
Interruptibility of workflow	Yes, after filtration.	Yes, after centrifugation.	Yes, after centrifugation and filtration.
Observed technical issues during extraction	Wastewater with high turbidity can cause clogging of the filters	Supernatant cannot be easily completely separated from the pellet	The supernatant cannot be easily separated from the pellet. However, because the supernatant is subsequently filtered, the solid and liquid phases are well separated.

For more details, see Supplementary Table S1. Manual workflow steps: Number of distinct operator actions required to complete the extraction workflow. Sample transfer steps: Number of times the sample must be transferred between tubes, filters, or vessels. Pipetting complexity: Qualitative estimate of pipetting workload considering volume range, number of pipetting steps, and precision requirements. (Low, High, Moderate)**.** Cross-contamination risk: Subjective assessment based on open handling steps and number of transfers. (Low, High, Moderate). Sample-loss risk: Estimated probability of losing material during filtration, pellet resuspension, or transfers. (Low, High, Moderate) Interruptibility: Whether the workflow can be paused without compromising the extraction process.

## Data Availability

The original contributions presented in this study are included in the article. Further inquiries can be directed to the corresponding author.

## Supplementary Materials

The following supporting information can be downloaded at https://www.mdpi.com/article/10.3390/microorganisms14102316/s1, Figure S1: CDF functions of sul1, tetW, blaOXA-48, blaVIM-1, vanA, and qnrS with the estimated mean (μ) and estimated standard deviation (σ). The intersection of the two red lines indicates the LoQ; Figure S2: Box plots of the recovery rates (%) of the biological replicates from the three methods being compared. Biological replicates spiked with *E. coli*. The black line is the median and the red line is the mean; Figure S3: Proportion of iDNA (solid phase) and exDNA (liquid phase) relative to the total ARG concentration, compared across the three methods examined. The following target genes are shown: (A) blaOXA-48 group, (B) blaVIM-1 Group, (C) sul1, (D) vanA, (E) QnrS1, (F) intl1, (G) tetW; Figure S4: Boxplots as logarithms of ARG concentration (GC/mL) (blaOXA-48 Group, sul1, blaVIM-1 Group, vanA, QnrS1, intl1, and tetW) from biological replicates (n = 11 iDNA; n = 10 exDNA) using combined method at measurement time t0. The median, 10th and 90th percentiles are shown as whiskers and minimum and maximum values as black dots. Further statistical metrics are presented in Table S7 supplement; Figure S5: Jittered box plots of coefficients of variation [%] of ARG targets (blaOXA-48 Group, sul1, blaVIM-1 Group, vanA, QnrS1, intl1, and tetW) between biological replicates of the different fractions (iDNA and exDNA) of combined method; Figure S6: Relative recovery of ARG concentrations in the iDNA-enriched and exDNA-enriched fractions during storage at 5 °C and −20 °C. The rows represent the ARG target genes sul1, tetW, and qnrS1, whilst the columns represent the two storage temperatures (yellow = 5 °C, purple = −20 °C). The relative recovery [%] was calculated for each observation as the ratio of the concentration at the storage time point t to the corresponding target- and fraction-specific mean of the concentration at t_0_. Individual observations, mean at each time point are presented. The common t0 reference comprised replicates for iDNA (n = 11) and for exDNA (n = 10); t1–t9 each comprised two replicates per fraction and storage temperature. The purple (iDNA) and blue (exDNA) horizontal lines indicate the target- and fraction-specific limits for the maximum allowable deviation (MAD); Table S1: Information about dPCR Assays with Target, target region, GeneGlobe Catalog number and manufacturer information; Table S2: Summary usability, operational time, qualitative workflow and ressource observation; Table S3: coefficients of variation [%], minimum and maximum of ARG concentration [GC/mL] of the targets (blaOXA-48 group, sul1, blaVIM-1 group, vanA, QnrS1, intl1, and tetW) for iDNA and exDNA fraction between biological replicates for the combined method; Table S4: Cost estimate for filtration method. Costs per biological sample replicate for consumables and reagents for the comparative tests in the wastewater monitoring laboratory at Hamm Lippstadt University of Applied Sciences without subsequent dPCR analysis (based on prices in 2024); Table S5: Cost estimate for centrifugation method. Costs per biological sample replicate for consumables and reagents for the comparative tests in the wastewater monitoring laboratory at Hamm Lippstadt University of Applied Sciences without subsequent dPCR analysis (based on prices in 2024); Table S6: Cost estimate for combinded method. Costs per biological sample replicate for consumables and reagents for the comparative tests in the wastewater monitoring laboratory at Hamm Lippstadt University of Applied Sciences without subsequent dPCR analysis (based on prices in 2024); Table S7: Endogenous and spiked total Escherichia coli concentrations, spike-to-background ratios, and descriptive whole-process recoveries for the three wastewater DNA fractionation methods; Table S8: ARG concentration and iDNA and exDNA proportions [% of the targets (blaOXA-48 group, sul1, blaVIM-1 group, vanA, QnrS1, intl1, and tetW) for the centrifugation, filtration, and combined methods; Table S9: Target- and fraction-specific limits for the maximum allowable deviation (MAD).

## References

[1] European Antimicrobial Resistance Collaborators (2022). The burden of bacterial antimicrobial resistance in the WHO European region in 2019: A cross-country systematic analysis. Lancet Public Health.

[2] WHO Regional Office for Europe (2022). A Health Perspective on the Role of The Environment in One Health.

[3] Pazda M., Kumirska J., Stepnowski P., Mulkiewicz E. (2019). Antibiotic resistance genes identified in wastewater treatment plant systems—A review. Sci. Total Environ..

[4] Cacace D., Fatta-Kassinos D., Manaia C.M., Cytryn E., Kreuzinger N., Rizzo L., Karaolia P., Schwartz T., Alexander J., Merlin C. (2019). Antibiotic resistance genes in treated wastewater and in the receiving water bodies: A pan-European survey of urban settings. Water Res..

[5] Woegerbauer M., Bellanger X., Merlin C. (2020). Cell-Free DNA: An Underestimated Source of Antibiotic Resistance Gene Dissemination at the Interface Between Human Activities and Downstream Environments in the Context of Wastewater Reuse. Front. Microbiol..

[6] Zhang X.-X., Zhang T., Fang H.H.P. (2009). Antibiotic resistance genes in water environment. Appl. Microbiol. Biotechnol..

[7] Guo J., Li J., Chen H., Bond P.L., Yuan Z. (2017). Metagenomic analysis reveals wastewater treatment plants as hotspots of antibiotic resistance genes and mobile genetic elements. Water Res..

[8] Savin M., Hammerl J.A., Hassa J., Hembach N., Kalinowski J., Schwartz T., Droop F., Mutters N.T. (2023). Free-floating extracellular DNA (exDNA) in different wastewaters: Status quo on exDNA-associated antimicrobial resistance genes. Environ. Pollut..

[9] Tiwari A., Radu E., Kreuzinger N., Ahmed W., Pitkänen T. (2024). Key considerations for pathogen surveillance in wastewater. Sci. Total Environ..

[10] Ajayi A.O., Odeyemi A.T., Akinjogunla O.J., Adeyeye A.B., Ayo-Ajayi I. (2024). Review of antibiotic-resistant bacteria and antibiotic resistance genes within the one health framework. Infect. Ecol. Epidemiol..

[11] European Parliament, Council of the European Union (2024). Directive (EU) 2024/3019 of the European Parliament and of the Council of 27 November 2024 Concerning Urban Wastewater Treatment (Recast).

[12] Zarei-Baygi A., Smith A.L. (2021). Intracellular versus extracellular antibiotic resistance genes in the environment: Prevalence, horizontal transfer, and mitigation strategies. Bioresour. Technol..

[13] Sharma V.K., Johnson N., Cizmas L., McDonald T.J., Kim H. (2016). A review of the influence of treatment strategies on antibiotic resistant bacteria and antibiotic resistance genes. Chemosphere.

[14] Calderón-Franco D., van Loosdrecht M.C.M., Abeel T., Weissbrodt D.G. (2021). Free-floating extracellular DNA: Systematic profiling of mobile genetic elements and antibiotic resistance from wastewater. Water Res..

[15] Sivalingam P., Poté J., Prabakar K. (2020). Extracellular DNA (eDNA): Neglected and Potential Sources of Antibiotic Resistant Genes (ARGs) in the Aquatic Environments. Pathogens.

[16] Sivalingam P., Sabatino R., Sbaffi T., Fontaneto D., Corno G., Di Cesare A. (2023). Extracellular DNA includes an important fraction of high-risk antibiotic resistance genes in treated wastewaters. Environ. Pollut..

[17] Zhang Y., Li A., Dai T., Li F., Xie H., Chen L., Wen D. (2018). Cell-free DNA: A Neglected Source for Antibiotic Resistance Genes Spreading from WWTPs. Environ. Sci. Technol..

[18] Sui Q., Chen Y., Yu D., Wang T., Hai Y., Zhang J., Chen M., Wei Y. (2019). Fates of intracellular and extracellular antibiotic resistance genes and microbial community structures in typical swine wastewater treatment processes. Environ. Int..

[19] Li F., Liu K., Bao Y., Li Y., Zhao Z., Wang P., Zhan S. (2024). Molecular level removal of antibiotic resistant bacteria and genes: A review of interfacial chemical in advanced oxidation processes. Water Res..

[20] Erler T., Droop F., Lübbert C., Knobloch J.K., Carlsen L., Papan C., Schwanz T., Zweigner J., Dengler J., Hoffmann M. (2024). Analysing carbapenemases in hospital wastewater: Insights from intracellular and extracellular DNA using qPCR and digital PCR. Sci. Total Environ..

[21] Tiwari A., Kurittu P., Al-Mustapha A.I., Heljanko V., Johansson V., Thakali O., Mishra S.K., Lehto K.-M., Lipponen A., Oikarinen S. (2022). Wastewater surveillance of antibiotic-resistant bacterial pathogens: A systematic review. Front. Microbiol..

[22] Slipko K., Reif D., Schaar H., Saracevic E., Klinger A., Wallmann L., Krampe J., Woegerbauer M., Hufnagl P., Kreuzinger N. (2022). Advanced wastewater treatment with ozonation and granular activated carbon filtration: Inactivation of antibiotic resistance targets in a long-term pilot study. J. Hazard. Mater..

[23] Keenum I., Liguori K., Calarco J., Davis B.C., Milligan E., Harwood V.J., Pruden A. (2022). A framework for standardized qPCR-targets and protocols for quantifying antibiotic resistance in surface water, recycled water and wastewater. Crit. Rev. Environ. Sci. Technol..

[24] Hock L., Luiken R., Valério E., Vargha M., Vierheilig J., Börjesson S., Pitkänen T., Schmitt H. (2026). Integrating AMR surveillance into wastewater monitoring systems in 2025: A position on the implementation of Article 17 of the Urban Wastewater Treatment Directive (UWWTD). Euro Surveill..

[25] Wang S., Ma X., Liu Y., Yi X., Du G., Li J. (2020). Fate of antibiotics, antibiotic-resistant bacteria, and cell-free antibiotic-resistant genes in full-scale membrane bioreactor wastewater treatment plants. Bioresour. Technol..

[26] Li A.-D., Metch J.W., Wang Y., Garner E., Zhang A.N., Riquelme M.V., Vikesland P.J., Pruden A., Zhang T. (2018). Effects of sample preservation and DNA extraction on enumeration of antibiotic resistance genes in wastewater. FEMS Microbiol. Ecol..

[27] Davis B.C., Brown C., Gupta S., Calarco J., Liguori K., Milligan E., Harwood V.J., Pruden A., Keenum I. (2023). Recommendations for the use of metagenomics for routine monitoring of antibiotic resistance in wastewater and impacted aquatic environments. Crit. Rev. Environ. Sci. Technol..

[28] Gillings M.R., Gaze W.H., Pruden A., Smalla K., Tiedje J.M., Zhu Y.-G. (2015). Using the class 1 integron-integrase gene as a proxy for anthropogenic pollution. ISME J..

[29] Storteboom H., Arabi M., Davis J.G., Crimi B., Pruden A. (2010). Tracking antibiotic resistance genes in the South Platte River basin using molecular signatures of urban, agricultural, and pristine sources. Environ. Sci. Technol..

[30] Mazel D. (2006). Integrons: Agents of bacterial evolution. Nat. Rev. Microbiol..

[31] Schwermer C.U., Krzeminski P., Anglès d’Auriac M., Gjeitnes M., Moe J., Bellanger X., Bürgmann H., Carapeto García R., Crespo Iniesta P., Dagot C. (2025). Pilot Study on Antimicrobial Resistance Monitoring in European Surface Waters—Final Report of the Eionet Working Group.

[32] World Health Organization (2025). Wastewater and Environmental Surveillance Summary for Antimicrobial Resistance: Draft.

[33] Munk P., Brinch C., Møller F.D., Petersen T.N., Hendriksen R.S., Seyfarth A.M., Kjeldgaard J.S., Svendsen C.A., van Bunnik B., Berglund F. (2022). Genomic analysis of sewage from 101 countries reveals global landscape of antimicrobial resistance. Nat. Commun..

[34] (1983). German Standardised Method for the Analysis of Water, Waste Water and Sludge; Cations (Group E); Determination of Ammonium Nitrogen (E 5).

[35] (2011). German Standardised Methods for the Analysis of Water, Waste Water and Sludge-Anions (Group D)-Part 9: Photometric Determination of Nitrate (D 9).

[36] (2003). Water Quality—Determination of Chemical Oxygen Demand (ST-COD): Cuvette Test (ISO 15705:2002).

[37] Ficetola G.F., Miaud C., Pompanon F., Taberlet P. (2008). Species detection using environmental DNA from water samples. Biol. Lett..

[38] Bivins A., North D., Wu Z., Shaffer M., Ahmed W., Bibby K. (2021). Within- and between-Day Variability of SARS-CoV-2 RNA in Municipal Wastewater during Periods of Varying COVID-19 Prevalence and Positivity. ACS EST Water.

[39] Ahmed W., Smith W.J.M., Metcalfe S., Jackson G., Choi P.M., Morrison M., Field D., Gyawali P., Bivins A., Bibby K. (2022). Comparison of RT-qPCR and RT-dPCR Platforms for the Trace Detection of SARS-CoV-2 RNA in Wastewater. ACS EST Water.

[40] (2022). Water Quality, Sampling, Part 25: Guideline on the Validation of the Storage Time of Water Samples.

[41] Oliveira M., Nunes M., Barreto Crespo M.T., Silva A.F. (2020). The environmental contribution to the dissemination of carbapenem and (fluoro)quinolone resistance genes by discharged and reused wastewater effluents: The role of cellular and extracellular DNA. Water Res..

[42] Rocha J., Cacace D., Kampouris I., Guilloteau H., Jäger T., Marano R.B., Karaolia P., Manaia C.M., Merlin C., Fatta-Kassinos D. (2020). Inter-laboratory calibration of quantitative analyses of antibiotic resistance genes. J. Environ. Chem. Eng..

[43] Liguori K., Calarco J., Maldonado Rivera G., Kurowski A., Keenum I., Davis B.C., Harwood V.J., Pruden A. (2023). Comparison of Cefotaxime-Resistant Escherichia coli and sul1 and intI1 by qPCR for Monitoring of Antibiotic Resistance of Wastewater, Surface Water, and Recycled Water. Antibiotics.

[44] Chen Y.-C., Higgins M.J., Maas N.A., Murthy S.N. (2006). DNA extraction and Escherichia coli quantification of anaerobically digested biosolids using the competitive touchdown PCR method. Water Res..

[45] Zöhrer J., Ascher-Jenull J., Wagner A.O. (2025). Tracking Different States of Spiked Environmental DNA Using Multiplex Digital PCR Assays. Environ. Microbiol..

[46] Fretschner T., Zeisler E.E., Scheller S., Aurenz A., Schneider B., Lukas M., Braun U., Greiner T., Schumacher J., Selinka H.C. (2025). Comparative assessment of combined concentration and extraction methods for Influenza A and B virus detection in wastewater. Front. Environ. Sci..

[47] Yuan Q.-B., Huang Y.-M., Wu W.-B., Zuo P., Hu N., Zhou Y.-Z., Alvarez P.J.J. (2019). Redistribution of intracellular and extracellular free & adsorbed antibiotic resistance genes through a wastewater treatment plant by an enhanced extracellular DNA extraction method with magnetic beads. Environ. Int..

[48] Zhang S., Medina J.S., Wang T., Augsburger N., Al-Gashgari B., Hong P.-Y. (2024). Extracting cell-free extracellular DNA from wastewater: Evaluation of extraction protocols for use in complex matrices. J. Hazard. Mater. Adv..

[49] Vuillemin A., Horn F., Alawi M., Henny C., Wagner D., Crowe S.A., Kallmeyer J. (2017). Preservation and Significance of Extracellular DNA in Ferruginous Sediments from Lake Towuti, Indonesia. Front. Microbiol..

[50] Mao D., Luo Y., Mathieu J., Wang Q., Feng L., Mu Q., Feng C., Alvarez P.J.J. (2014). Persistence of extracellular DNA in river sediment facilitates antibiotic resistance gene propagation. Environ. Sci. Technol..

[51] Wells J.E., Durso L.M., Ibekwe A.M., Frye J.G., Sharma M., Williams C.F., Shamimuzzaman M. (2025). Agriculturally Sourced Multidrug-Resistant Escherichia coli for Use as Control Strains. Pathogens.

[52] Mikel P., Vasickova P., Tesarik R., Malenovska H., Kulich P., Vesely T., Kralik P. (2016). Preparation of MS2 Phage-Like Particles and Their Use As Potential Process Control Viruses for Detection and Quantification of Enteric RNA Viruses in Different Matrices. Front. Microbiol..

[53] Nguyen A.Q., Vu H.P., Nguyen L.N., Wang Q., Djordjevic S.P., Donner E., Yin H., Nghiem L.D. (2021). Monitoring antibiotic resistance genes in wastewater treatment: Current strategies and future challenges. Sci. Total Environ..

[54] Pham V.T., Harris-Ryden Z., Kodali N.S., Hamner T.F., Popalzai S., Hernandez M.C., Ajose M., Owens M., Riedel T.E., Reichler S. (2025). Filter, heat, spin: A simple and inexpensive method for DNA preparation from freshwater for use in high-throughput molecular source tracking. PLoS Water.

[55] Gao M., Kortum P., Oswald F.L. (2020). Multi-Language Toolkit for the System Usability Scale. Int. J. Hum.–Comput. Interact..

[56] Klug B. (2017). An Overview of the System Usability Scale in Library Website and System Usability Testing. Weav. J. Libr. User Exp..

[57] Bangor A., Kortum P.T., Miller J.T. (2008). An Empirical Evaluation of the System Usability Scale. Int. J. Hum.–Comput. Interact..

[58] Peres S.C., Pham T., Phillips R. (2013). Validation of the System Usability Scale (SUS). Proc. Hum. Factors Ergon. Soc. Annu. Meet..

[59] Calero-Cáceres W., Muniesa M. (2016). Persistence of naturally occurring antibiotic resistance genes in the bacteria and bacteriophage fractions of wastewater. Water Res..

[60] OW, US EPA, Office of Science and Technology (2013). Method 1609: Enterococci in Water by TaqMan^®^ Quantitative Polymerase Chain Reaction (qPCR) with Internal Amplification Control (IAC) Assay: EPA-820-R-13-005.

[61] Gilpin B.J., Devane M., Nourozi F., Robson B., Scholes P., Lin S. (2013). Recommendations for the processing and storage of water samples before polymerase chain reaction (PCR) analysis. N. Z. J. Mar. Freshw. Res..

[62] Hinlo R., Gleeson D., Lintermans M., Furlan E. (2017). Methods to maximise recovery of environmental DNA from water samples. PLoS ONE.

[63] Tavazzi S., Cacciatori C., Comero S., Fatta-Kassinos D., Karaolia P., Iakovides I.C., Loutsiou P., Gutierrez-Aguirre I., Lengar Z., Bajde I. (2023). Short-term stability of wastewater samples for storage and shipment in the context of the EU Sewage Sentinel System for SARS-CoV-2. J. Environ. Chem. Eng..

[64] Sedlmayr I., Schenekar T. (2024). eDNA State and Medium Affect DNA Degradation Patterns in Seminatural Systems of Southern African Waterholes. Environ. DNA.

[65] Mauvisseau Q., Harper L.R., Sander M., Hanner R.H., Kleyer H., Deiner K. (2022). The Multiple States of Environmental DNA and What Is Known about Their Persistence in Aquatic Environments. Environ. Sci. Technol..

[66] Deng P., Hu X., Cai W., Zhang Z., Zhang Y., Huang Y., Yang Y., Li C., Ai S. (2023). Profiling of intracellular and extracellular antibiotic resistance genes in municipal wastewater treatment plant and their effluent-receiving river. Environ. Sci. Pollut. Res..

[67] Wang R., Mardalisa, Hara-Yamamura H., Matsuura N., Honda R. (2025). Applicability of intI1 as an indicator gene for securing the removal efficiency of extracellular antimicrobial resistance genes in full-scale wastewater treatment plants. Bioresour. Technol..

[68] Pärnänen K.M.M., Narciso-da-Rocha C., Kneis D., Berendonk T.U., Cacace D., Do T.T., Elpers C., Fatta-Kassinos D., Henriques I., Jaeger T. (2019). Antibiotic Resistance in European Wastewater Treatment Plants Mirrors the Pattern of Clinical Antibiotic Resistance Prevalence.

[69] Li J., Ren S., Qiu X., Zhao S., Wang R., Wang Y. (2022). Electroactive Ultrafiltration Membrane for Simultaneous Removal of Antibiotic, Antibiotic Resistant Bacteria, and Antibiotic Resistance Genes from Wastewater Effluent. Environ. Sci. Technol..

[70] Liguori K., Keenum I., Davis B.C., Calarco J., Milligan E., Harwood V.J., Pruden A. (2022). Antimicrobial Resistance Monitoring of Water Environments: A Framework for Standardized Methods and Quality Control. Environ. Sci. Technol..

