# Supplementary material for: Combined Detection of Intracellular and Extracellular Antibiotic Resistance Genes in Municipal Wastewater—Sample Preparation Methods and Storage Stability
Source: Microorganisms. 2026 Oct 10;14(10):2316. doi: 10.3390/microorganisms14102316 (PMC13707453; doi:10.3390/microorganisms14102316)
Supplement: Supplementary file 1 [file microorganisms-14-02316-s001.zip › microorganisms-4432181-supplementary.pdf]

Combined Detection of Intracellular and Extracellular Antibiotic Resistance Genes in Municipal Wastewater—Sample Preparation Methods and Storage Stability

Sarah Boeker-Haeusser [1,2], Hanna Wrede [1], Claudia Klümper [1,*] and Silvio Beier [2]

1 Hamm-Lippstadt University of Applied Sciences, Department Hamm 2, 59063 Hamm, Germany
2 Bauhaus-Institute for Infrastructure Solutions (b.is), Bauhaus University Weimar, 99423 Weimar, Germany

Supplement

A comprehensive questionnaire designed to evaluate the user-friendliness of systems, with reference to the framework established by Gao et al. [1]:

General information

Operator: ______________________

Sample: ______________________

Extraction method:

☐ Filtration ☐ Centrifugation ☐ Combined method

Date: ______________________

Please indicate how strongly you agree with the following statements.

Scale:

1 = Strongly disagree 2 = Disagree 3 = Neutral 4 = Agree 5 = Strongly agree

No	Statement	1	2	3	4	5
1	I would like to use this method frequently in the laboratory.					
2	I found the method unnecessarily complex.					
3	I thought the method was easy to use.					
4	I think I would need assistance from a technically experienced person to perform this method.					
5	The different steps of the workflow were well integrated.					
6	I thought there was too much inconsistency in the workflow.					
7	I imagine that most people would learn to perform this method very quickly.					
8	I found the method cumbersome or awkward to use.					
9	I felt confident performing this method.					
10	I needed to learn a lot of things before I could perform this method.					

Information about primer probe Assays

For detection of *sul1*, *bla*$_{VIM1}$ group, *bla*$_{OXA-48}$ group, *vanA, E.coli,QnrS1* primer/probe Assays (QIAGEN GmbH, Hilden, Germany), and of ARG *tetW* and *intl1* GT-Digital intl1;tetW Assay Kit for the QIAcuity® Digital PCR System (GT-Digital intl1;tetW Assay Kit for the QIAcuity® Digital PCR System, Fort Collins, Colorado, USA) the primer /probe Assays in Table S1 was used according to the manufacturer's protocol.

Table S1: *Information about dPCR Assays with Target, target region, GeneGlobe Catalog number and manufacturer information.*

Target	Target region	GeneGlobe Catalog number	Manufacturer	Manufacturer number
Intl1; tetW Duplex Assay Kit	No information from customer	-	GT Molecular	100549
Sul1	Bacterial quaternary ammonium compound efflux SMR transporter QacE delta 1 gene	DMA00587-H	QIAGEN	250207
bla$_{VIM-1}$group	Bacterial subclass B1 metallo-beta-lactamase VIM1 gene ; Class B beta-lactamase	DMA00528-R	QIAGEN	250207
vanA (VRE)	Bacterial D-alanine--(R)-lactate ligase VanA gene	DMA00578-C	QIAGEN	250207
qnrS1	Bacterial quinolone resistance pentapeptide repeat protein QnrS1 gene ; Fluoroquinolone resistance	DMA00566-T	QIAGEN	250207
E.coli	16S ribosomal RNA gene	DMA00140-F	QIAGEN	250207
bla$_{OXA-48}$ group	Bacterial OXA-48 family class D beta-lactamase OXA-1055 gene ; Class D beta-lactamase	DMA00548-F	QIAGEN	250207

Analytical LoQ dPCR

Quantification of ARG copy numbers in technical dPCR replicates along a 1:10 dilution series of DNA positive control material. A Gaussian distribution fitted to the proportion of positive technical replicates along the dilution series and used to estimate the 95% LOQ per µl [2] .

CDF functions of *sul1*, *tetW*, *bla*$_{\text{OXA-48}}$ group, *bla*$_{\text{VIM-1}}$ group, *vanA* and *QnrS1* with the indication of the estimated mean (µ) and the estimated standard deviation (σ). The intersection of the two red lines shows the LoQ.

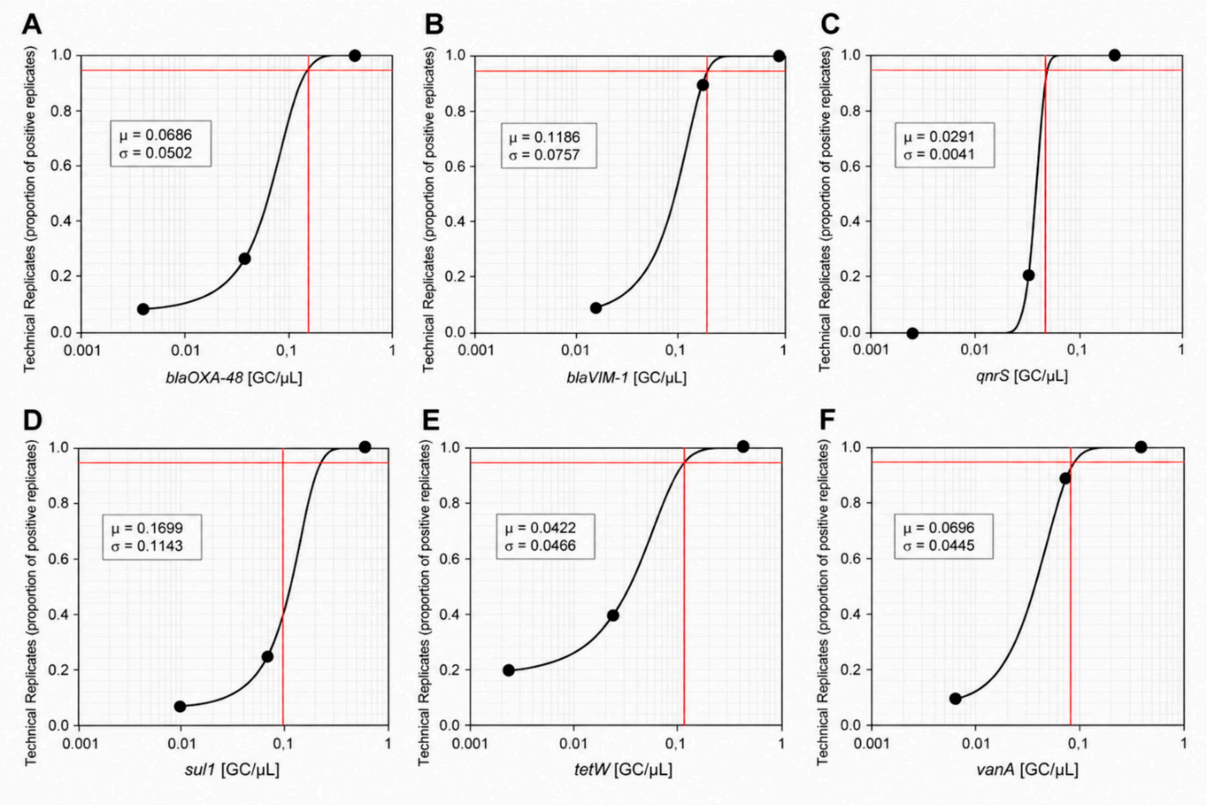

Figure S1: *CDF functions of sul1, tetW, blaOXA-48, blaVIM-1, vanA, and qnrS with the estimated mean (µ) and estimated standard deviation (σ). The intersection of the two red lines indicates the LoQ.*

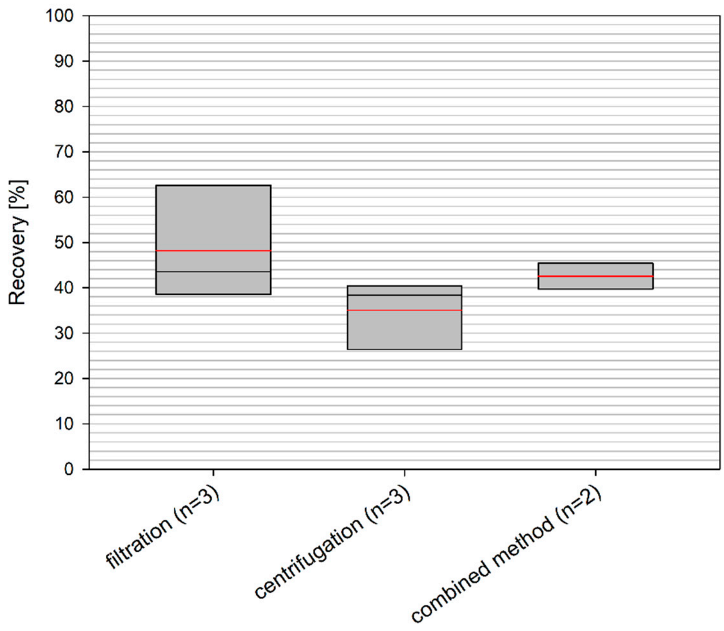

Figure S2: *Box plots of the recovery rates (%) of the biological replicates from the three methods being compared. Biological replicates spiked with E.coli. The black line is the median and the red line is the mean.*

Preparation of the *E. coli* spike

The *E. coli* spike is generated by growing a pure *E. coli* culture (DSM 1103 / ATCC 25922 *Escherichia coli* (Migula 1895) Castellani and Chalmers 1919; Leibniz Institute DSMZ-German Collection of Microorganisms and Cell Cultures GmbH) in Luria-Bertani medium (LB medium) . To produce the E. coli spike, 50 mL of LB medium is added to an Erlenmeyer flask and 0.5 mL of the pure E. coli culture (DSM 1103) is inoculated. The Erlenmeyer flask is then incubated in a shaking incubator at 37 °C and 100 rpm (rounds per minute). To check the growth of the *E. coli* culture, the optical density at 600 nm (OD600) is measured every 30 minutes using the photometer (device name, manufacturer). To measure the OD600, 1 mL of the *E. coli* culture is pipetted into 1 cm semi-micro cuvettes . The incubation is carried out until an OD600 of approximately 0.4 is reached . The *E. coli* culture stored on ice.

To quantify the *E. coli* bacteria in the bacterial spike suspension, the DNA is extracted from 1.5 mL of the *E. coli* suspension by using the Monarch® Genomic DNA Purification Kit (New England Biolabs GmbH). The recovery rate of the kit is between 80 % and 85 %. The assigned spike concentration was corrected assuming an extraction recovery of 85%. Then the DNA extract is quantified using digital PCR system (QIAcuity One, 5plex Instrument, QIAGEN GmbH, Hilden, Germany) with QIAcuity Nanoplate 8.5k dPCR plates (QIAcuity Nanoplate 8.5k 24-Well, QIAGEN GmbH, Hilden, Germany). For *E. coli*, a commercial primer/probe assay DMA00140-F (QIAGEN GmbH, Hilden, Germany) was used. No-template control (NTC) and positive control for *E.coli* from Qiagen Microbiota Control V2 (QIAGEN GmbH, Hilden, Germany) were also analysed to exclude contamination and check the correct performance of the digital PCR. The digital PCR-assays were set up in a reaction volume of 12 µl,

including 3 µl nucleic acid extract, 0.6 µl 20x *E.coli* primer probe mix, 3 µl QIAcuity Probe PCR Mastermix and 5.4 µl nuclease-free water. Cycling conditions for the digital PCR assay were as follows: polymerase activation at 95 °C for 2 min and 40 cycles of denaturation, annealing/extension at 95 °C/15 s, then 62 °C/60 s, respectively. For storage, the *E. coli* suspension is mixed with glycerol and frozen at -80 °C.

Relative contribution of iDNA (solid phase) and exDNA (liquid phase) to total DNA (mean percentages) using filtration, centrifugation and a combined method:

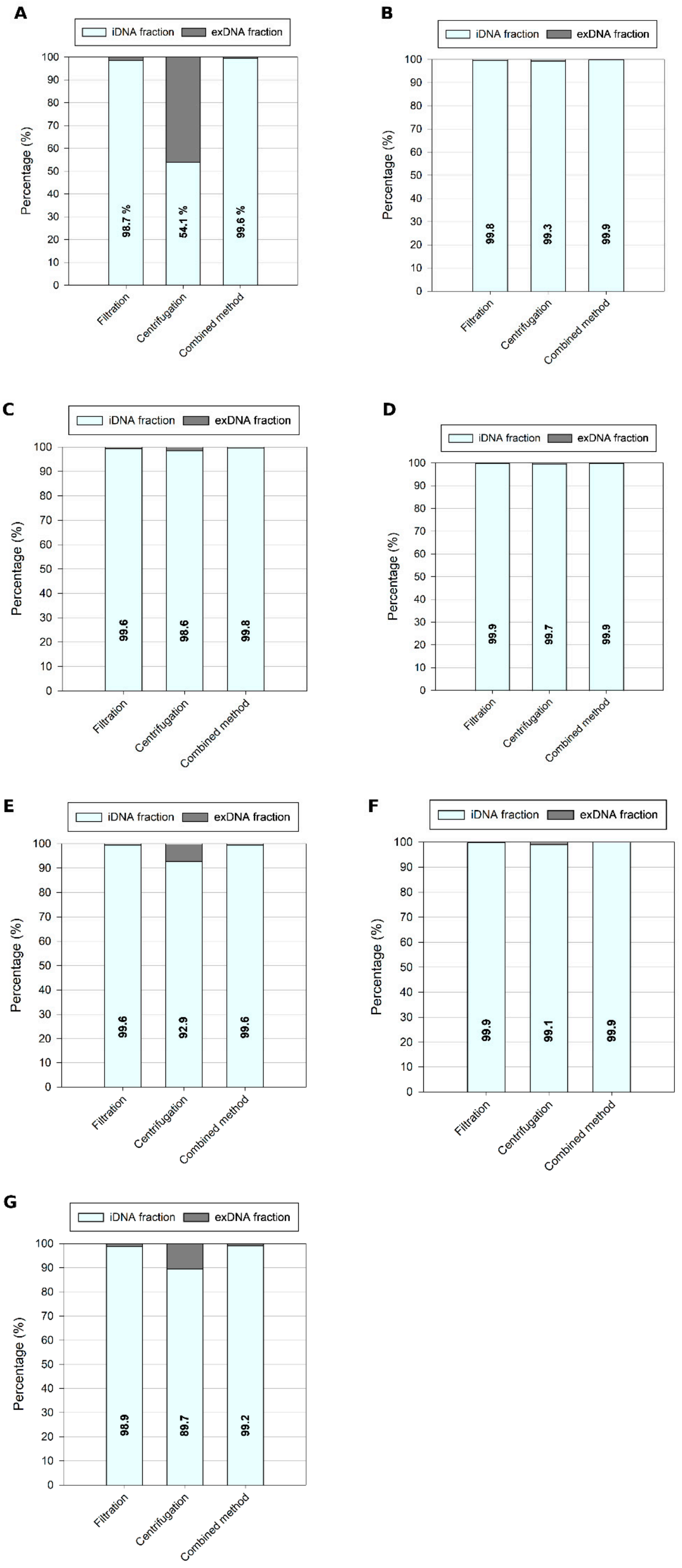

Figure S3: *Proportion of iDNA (solid phase) and exDNA (liquid phase) relative to the total ARG concentration, compared across the three methods examined. The following target genes are shown: (A) blaOXA-48 group, (B) blaVIM-1 Group, (C) sul1, (D) vanA, (E) QnrS1, (F) intl1, (G) tetW.*

Handling characteristics of filtration, centrifugation and combined method

Table S2: *Summary usability, operational time, qualitative workflow and ressource observation.*

	Filtration	Centrifugation	Combined method
**Time-in-motion**			
hands-on time (HoT)	3:15 h by two operators	2:45 h by two operators	3:00 h by two operators
total turnaround time (TaT)	6 h by two operators	5 h by two operators	6 h by two operators
run-associated cleaning/decontamination hands-on time	4 h, including autoclaving and sterilization of filter cartridge and Maxwell® RSC Instrument decontamination program	1.5 h, including Maxwell® RSC Instrument decontamination program	4 h, including autoclaving and sterilization of filter cartridge and Maxwell® RSC Instrument decontamination program
**System Usability Scale (SuS)**	72.5	60	72.5
**overhead**			
required equipment inventory	QMR 25 homogenizer, pressure filter unit (200 ml), centrifuge and rotor for 10,000 g at 2 ml volume, heating block, eluator vacuum manifold, FastPrep-24™ 5G Homogenizer, vacuum and compressed air line or pump, Maxwell RSC device, micropipettes	QMR 25 homogenizer, centrifuge and rotor for 10,000 g at 2 mL volume and rotor for 10,000 g for 50 mL volume, heating block, eluator vacuum manifold, FastPrep-24™ 5G Homogenizer, vacuum air line or pump, Maxwell RSC device, micropipettes	QMR 25 homogenizer, pressure filter unit (200 ml), centrifuge and rotor for 10,000 g at 2 mL volume and rotor for 10,000 g for 50 mL volume, heating block, eluator vacuum manifold, FastPrep-24™ 5G Homogenizer, vacuum and compressed air line or pump, Maxwell RSC device, micropipettes
**resource**			
required qualification	Routine laboratory staff/scientists or laboratory staff after training	Routine laboratory staff/scientists or laboratory staff after training	Routine laboratory staff/scientists or laboratory staff after training
infrastructure	Laboratory safety level 1, spatially separated areas between dPCR analysis and sample extraction, compressed air line,	Laboratory safety level 1, spatially separated areas between dPCR analysis and sample extraction.	Laboratory safety level 1, spatially separated areas between dPCR analysis and sample extraction, compressed air line,

	vacuum air line or pump		vacuum air line or pump
**Workflow parameter**			
Number of operators	2	2	2
Pipetting complexity (low / moderate / high)	Moderate	high	moderate
Risk of cross-contamination between liquid and solid phase (low / moderate / high)	low	high	Low
Risk of sample loss during processing (low / moderate / high)	moderate	high	Moderate
Risk of filter clogging	high	n/a	Low
Pellet handling required	n/a	Yes	Yes
Interruptibility of workflow	Yes, after filtration. Filters can be stored at -20 or 4 degrees Celsius. Filtrate must be analyses promptly.	Yes, after centrifugation. Pellets can be stored at -20 or 4 degrees Celsius. Supernatant must be analyses promptly.	Yes, after centrifugation and filtration. Pellets and filters can be stored at -20 or 4 degrees Celsius. Filtrate must be analyses promptly.
Observed technical issues during extraction	Wastewater with high turbidity can cause clogging of the filters	supernatant cannot be easily completed separated from the pellet	The supernatant cannot be easily separated from the pellet, but since the supernatant is subsequently filtered, it is ensured that the solid and liquid phases are well separated from each other.

Manual workflow steps: Number of distinct operator actions required to complete the extraction workflow.
Sample transfer steps: Number of times the sample must be transferred between tubes, filters, or vessels.
Pipetting complexity: Qualitative estimate of pipetting workload considering volume range, number of pipetting steps, and precision requirements.
Cross-contamination risk: Subjective assessment based on open handling steps and number of transfers.
Sample loss risk: Estimated probability of losing material during filtration, pellet resuspension, or transfers.
Interruptibility: Whether the workflow can be paused without compromising the extraction process.

Table S3: *coefficients of variation [%], minimum and maximum of ARG concentration [GC/mL] of the targets (blaOXA-48 group, sul1, blaVIM-1 group, vanA, QnrS1, intl1, and tetW) for iDNA and exDNA fraction between biological replicates for the combined method.*

**Target**	**Fraction**	**Average concentration genome copies per mL [GC/mL])**	**Min [GC/mL])**	**Max [GC/mL])**	**CV [%]**
bla$_{OXA-48}$ group	iDNA (n= 11)	9.94 x 10^3	6.91 x 10^3	1.33 x 10^4	17.8
	exDNA (n=10)	8.01 x 10^0	4.40 x 10^0	2.00 x10^1	56.3
bla$_{VIM-1}$ Group	iDNA (n= 11)	9.88 x 10^2	5.53 x 10^2	1.42 x10^3	23.4
	exDNA (n=10) *8 biological replicates < LoQ, but positive partitions	7.42 x 10^-1	4.8 x 10^-1 (1/2 LoQ)	1.06 x 10^0	35.7
Sul1	iDNA (n= 11)	2.48 x 10^6	1.73 x 10^6	3.13 x 10^6	13.6
	exDNA (n=10)	1.17 x 10^3	7.56 x 10^2	2.77 x 10^3	50.4
vanA	iDNA (n= 11)	2.33 x 10^2	1.12 x 10^2	3.24 x 10^2	29.2
	exDNA (n=8) *2 biological replicates < LoQ, and no positive partitions **6 biological replicates < LoQ, and positive partitions	3.69 x 10-1	2.8 x 10^-1 (1/2 LoQ)	8.40 x 10^-1	46.3
QnrS1	iDNA (n= 11)	3.50 x 10^5	2.68 x 10^5	4.07 x 10^5	12.9
	exDNA (n=10)	2.81 x 10^2	1.85 x 10^2	5.44 x 10^2	37.5
intl1	iDNA (n= 11)	3.66 x 10^6	2.87 x 10^6	4.08 x 10^6	10.5
	exDNA (n=10)	7.91 x 10^2	5.50 x 10^2	1.76 x 10^3	45.7
tetW	iDNA (n= 11)	1.32 x 10^6	1.07x 10^6	1.47 x 10^6	10.0
	exDNA (n=10)	3.09 x 10^3	2.18 x 10^3	5.03 x 10^3	25.9

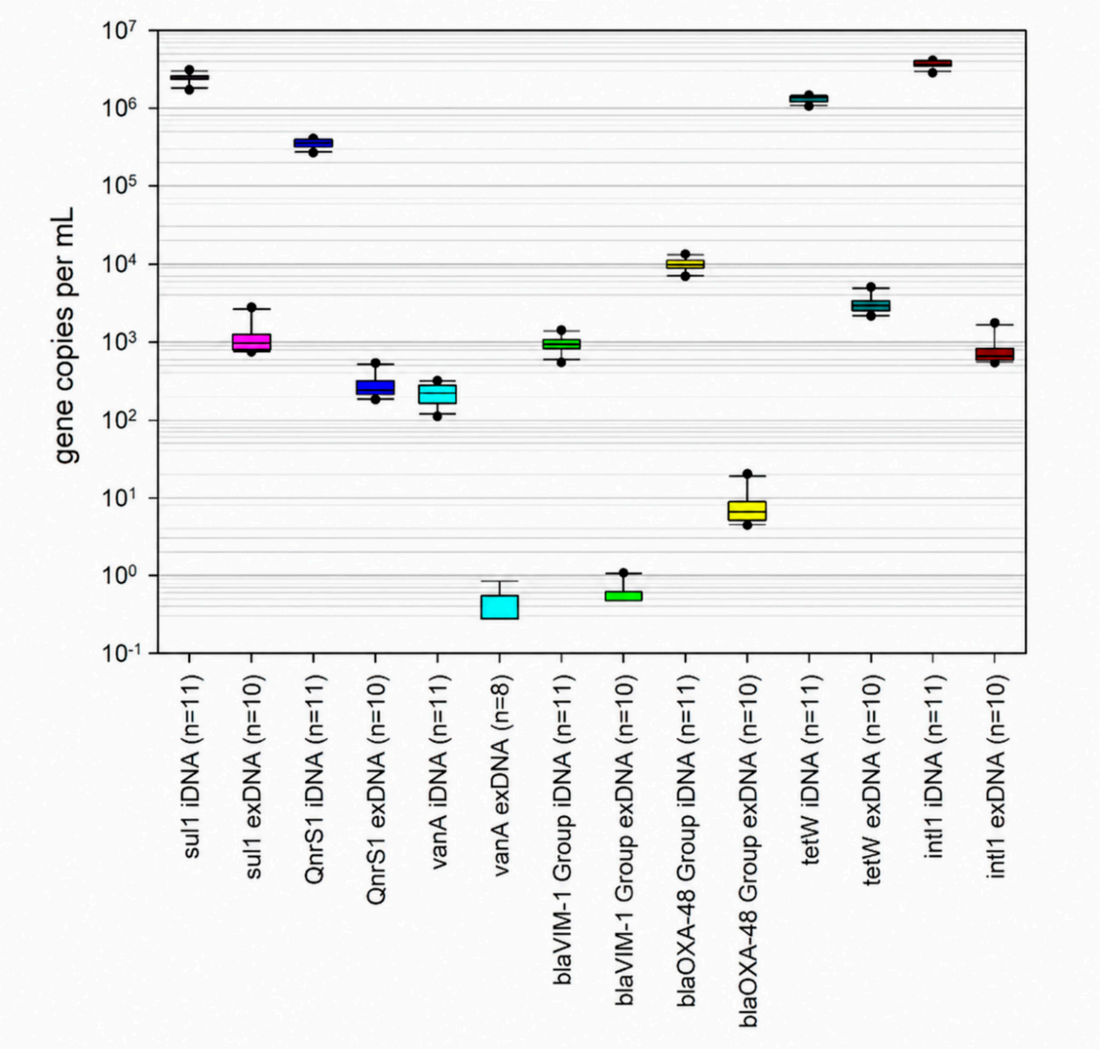

Figure S4: *Boxplots as logarithms of ARG concentration (GC/mL) (blaOXA-48 Group, sul1, blaVIM-1 Group, vanA, QnrS1, intl1, and tetW) from biological replicates (n = 11 iDNA; n = 10 exDNA) using combined method at measurement time t0. The median, 10th and 90th percentiles are shown as whiskers and minimum and maximum values as black dots.Further statistical metrics are presented in table S7 supplement.*

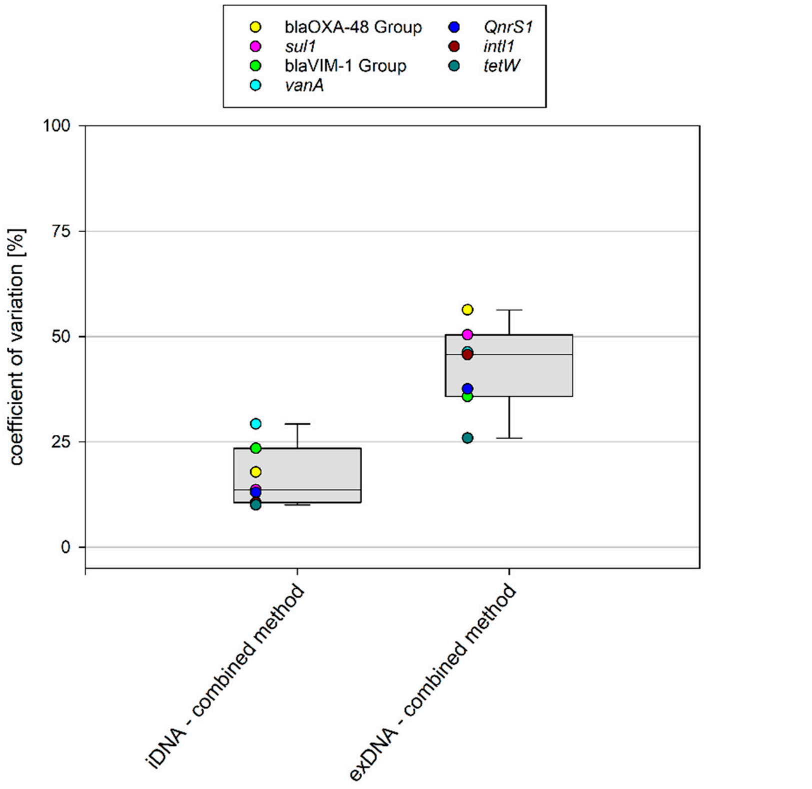

Figure S5: *Jittered box plots of coefficients of variation [%] of ARG targets (blaOXA-48 Group, sul1, blaVIM-1 Group, vanA, QnrS1, intl1, and tetW) between biological replicates of the different fractions (iDNA and exDNA) of combined method.*

Cost comparison for the filtration method, the centrifugation method, and the combined method.

Table S4: *Cost estimate for filtration method. Costs per biological sample replicate for consumables and reagents for the comparative tests in the wastewater monitoring laboratory at Hamm Lippstadt University of Applied Sciences without subsequent dPCR analysis (based on prices in 2024).*

Items	Purpose	Metric	Amount used per wastewater sample biological replicate	Unit Price € (Netto)	Cost per sample replicate	Notes
0.2 µm, 47 mm Whatman Filter NucleporeTrackEtch Membrane	Filtration	filters	1	108.00 €	1.08 €	
120 ml Steril cup	Filtration	cup	1	65.00 €	0.26 €	
FastDNA SPIN Kit for Soil	Extraction	Kit	1	597.00 €	5.97 €	
15 mL Tubes steril	Extraction	tubes	1	126.00 €	0.25 €	
Ethanol 99.9% p.a	Extraction	mL	1	66.00 €	0.07 €	100 ml per Kit (100 reactions)
Maxwell(R) RSC Enviro Total Nucleic Acid Kit	Extraction	Kit	1	824.00 €	17.17 €	80 ml (other volumen from Protease, BB1, BB2, Isopropanol per extraktion)
Protease	Extraction	mL	1	42.00 €	1.40 €	
Binding Buffer 1	Extraction	mL	24	143.00 €	10.73 €	
Binding Buffer 2	Extraction	mL	2	160.00 €	10.67 €	
Ethanol (95 % p.a)	Extraction	mL	0.14	66.00 €	0.01 €	per kit 350 mL
Isopropanol	Extraction	mL	96.84	92.00 €	3.56 €	57 mL and 96 mL per extraktion (80ml)
50 ml steril tubes	Extraction	tubes	2	161.00 €	0.64 €	
120 ml Steril cup	Extraction	cup	2	65.00 €	0.52 €	
1000 µL pipette tips with filter	Extraction	tips	3	82.00 €	0.26 €	
5 - 100 µL pipette tips with filter	Extraction	tips	1	69.00 €	0.07 €	
2.0 mL tubes	Extraction	tubes	1	61.70 €	0.06 €	
1.5 mL tubes	Extraction	tubes	3	45.20 €	0.14 €	
**Cost per biological sample replicate without dPCR**					**52.85 €**	

Table S5: *Cost estimate for centrifugation method. Costs per biological sample replicate for consumables and reagents for the comparative tests in the wastewater monitoring laboratory at Hamm Lippstadt University of Applied Sciences without subsequent dPCR analysis (based on prices in 2024).*

Items	Purpose	Metric	Amount used per wastewater sample biological replicate	Unit Price € (Netto)	Cost per sample replicate	Notes
FastDNA SPIN Kit for Soil	Extraction	Kit	1	597.00 €	5.97 €	
15 mL Tubes steril	Extraction	tubes	1	126.00 €	0.25 €	
Ethanol 99.9% p.a	Extraction	mL	1	66.00 €	0.07 €	100 ml per Kit (100 reactions)
Maxwell(R) RSC Enviro Total Nucleic Acid Kit	Extraction	Kit	1	824.00 €	17.17 €	80 ml (other volumen from Protease, BB1, BB2, Isopropanol per extraktion)
Protease	Extraction	mL	1	42.00 €	1.40 €	
Binding Buffer 1	Extraction	mL	24	143.00 €	10.73 €	
Binding Buffer 2	Extraction	mL	2	160.00 €	10.67 €	
Ethanol (95 % p.a)	Extraction	mL	0.14	66.00 €	0.01 €	per kit 350 mL
Isopropanol	Extraction	mL	96.84	92.00 €	3.56 €	57 mL and 96 mL per extraktion (80ml)
50 ml steril tubes	Extraction	tubes	4	161.00 €	1.29 €	
120 ml Steril cup	Extraction	cup	4	65.00 €	1.04 €	
1000 µL pipette tips with filter	Extraction	tips	5	82.00 €	0.43 €	
5 - 100 µL pipette tips with filter	Extraction	tips	1	69.00 €	0.07 €	
2.0 mL tubes	Extraction	tubes	1	61.70 €	0.06 €	
1.5 mL tubes	Extraction	tubes	3	45.20 €	0.14 €	
**Cost per biological sample replicate without dPCR**					**52.84 €**	

Table S6: *Cost estimate for combinded method. Costs per biological sample replicate for consumables and reagents for the comparative tests in the wastewater monitoring laboratory at Hamm Lippstadt University of Applied Sciences without subsequent dPCR analysis (based on prices in 2024).*

Items	Purpose	Metric	Amount used per wastewater sample biological replicate	Unit Price € (Netto)	Cost per sample replicate	Notes
0.2 µm, 47 mm Whatman Filter NucleporeTrackEtch Membrane	Filtration	filters	1	108.00 €	1.08 €	
120 ml Steril cup	Filtration	cup	1	65.00 €	0.26 €	
FastDNA SPIN Kit for Soil	Extraction	Kit	2	597.00 €	11.94 €	pellet and filter
15 mL Tubes steril	Extraction	tubes	2	126.00 €	0.50 €	
Ethanol 99,9% p.a	Extraction	mL	2	66.00 €	0.13 €	100 ml per Kit (100 reactions)
Maxwell(R) RSC Enviro Total Nucleic Acid Kit	Extraction	Kit	1	824.00 €	17.17 €	80 ml (other volumen from Protease, BB1, BB2, Isopropanol per extraktion)
Protease	Extraction	mL	1	42.00 €	1.40 €	
Binding Buffer 1	Extraction	mL	24	143.00 €	10.73 €	
Binding Buffer 2	Extraction	mL	2	160.00 €	10.67 €	
Ethanol (95 % p.a)	Extraction	mL	0.13	66.00 €	0.01 €	per kit 350 mL
Isopropanol	Extraction	mL	96.84	92.00 €	3.56 €	per kit 57 mL and 96 mL per extraktion
50 ml steril tubes	Extraction	tubes	4	161.00 €	1.29 €	
120 ml Steril cup	Extraction	cup	4	65.00 €	1.04 €	
1000 µL pipette tips with filter	Extraction	tips	5	82.00 €	0.43 €	
5 - 100 µL pipette tips with filter	Extraction	tips	1	69.00 €	0.07 €	
2.0 mL tubes	Extraction	tubes	1	61.70 €	0.06 €	
1.5 mL tubes	Extraction	tubes	3	45.20 €	0.14 €	
**Cost per biological sample replicate without dPCR**					**60.47 €**	

Table S7: *Endogenous and spiked total Escherichia coli concentrations, spike-to-background ratios, and descriptive whole-process recoveries for the three wastewater DNA fractionation methods.*

		endogenous *E. coli* concentration (gene copies / 2800 mL) per fraction	total endogenous *E. coli* concentration (gene copies / 2800 mL)	mean	SD	CV [%]	spike *E. coli* concentration (gene copies / 2800 mL)	total spike *E. coli* concentration (gene copies / 2800 mL)	mean	SD	CV [%]	spiked-to-background ratio (*)	nominal value spike *E. coli* concentration (gene copies / 2800 mL)	Recovery [%]
Filtration Method	M1	1.85x10^9	1.85x10^9	1.65x10^9	1.x10^8	10.9	9.01x10^9	9.03x10^9	1.12E+10	2.90x10^9	25.9	13.0 : 1	2.34x10^10	38
	Fi1	2.68x10^4					1.98x10^7							
	M2	1.50x10^9	1.50x10^9				1.00x10^10	1.00x10^10					2.30x10^10	43
	Fi2	1.07x10^5					7.23x10^6							
	M3	1.59x10^9	1.59x10^9				1.45x10^10	1.45x10^10					2.31x10^10	62
	Fi3	6.98x10^4					1.11x10^7							
Centrifugation Method	P1	1.13x10^9	1.18x10^9	1.14x10^9	1.23x10^8	10.8	6.93x10^9	8.60x10^9	7.85E+09	1.73x10^9	21.9	18.9 : 1	2.26x10^10	37
	Ü1	4.65x10^7					1.67x10^9							
	P2	9.35x10^8	9.97x10^8				4.52x10^9	5.87x10^9					2.25x10^10	26
	Ü2	6.16x10^7					1.35x10^9							
	P3	1.17x10^9	1.23x10^9				7.16x10^9	9.06x10^9					2.27x10^10	39
	Ü3	6.11x10^7					1.91x10^9							
Combined Method	P1	1.11x10^9	1.50x10^9	1.48x10^9	3.70x10^7	2.5	5.28x10^9	8.74x10^9	9.37E+09	8.87x10^8	9.4	14.6 : 1	2.27x10^10	38
	M1	3.98x10^8					3.44x10^9							
	Fi1	5.34x10^4					1.38x10^7							
	P2	9.73x10^8	1.45x10^9				6.49x10^9	9.99x10^9					2.26x10^10	44
	M2	4.77x10^8					3.46x10^9							
	Fi2	2.42x10^5					3.62x10^7							

*corrected concentration of the spike suspension = 2.1×10^{10} GC / 2800 mL

Table S8: *ARG concentration and iDNA and exDNA proportions [% of the targets (blaOXA-48 group, sul1, blaVIM-1 group, vanA, QnrS1, intl1, and tetW) for the centrifugation, filtration, and combined methods.*

**Target**	**Method**	**Total Average concentration genome copies per mL [GC/mL] (n= 3)**	**Percentage of iDNA [%]**	**Percentage of exDNA [%]**
bla_{OXA48} group	Filtration	1.2×10^3	98.6	1.3
	Centrifugation	2.3×10^2	78.1	21.9
	Combined method	1.3×10^3	99.5	0.5
bla_{VIM-1} group	Filtration	6.6×10^1	99.8	0.2
	Centrifugation	1.2×10^2	99.3	0.7
	Combined method	7.8×10^1	99.9	0.1
sul1	Filtration	3.7×10^5	99.6	0.4
	Centrifugation	7.2×10^5	98.7	1.3
	Combined method	4.7×10^5	99.8	0.2
vanA	Filtration	4.9×10^2	99.8	0.2
	Centrifugation	7.3×10^2	99.7	0.3
	Combined method	4.3×10^2	99.9	0.1
QnrS1	Filtration	1.5×10^5	99.6	0.4
	Centrifugation	1.2×10^5	92.9	7.1
	Combined method	8.9×10^4	99.6	0.4
intl1	Filtration	8.7×10^5	99.9	0.1
	Centrifugation	1.9×10^6	99.2	0.8
	Combined method	1.3×10^6	99.9	0.1
tetW	Filtration	7.9×10^5	99.0	1.0
	Centrifugation	1.1×10^6	90.0	10.0
	Combined method	8.8×10^5	99.3	0.7

Table S9: *Target- and fraction-specific limits for the maximum allowable deviation (MAD).*

target	iDNA lower MAD [%]	iDNA upper MAD [%]	exDNA lower MAD [%]	exDNA upper MAD [%]
tetW	83.7	116.6	59.9	140.1
qnrS1	80.1	119.9	41.8	158.2
Sul1	79.0	121.0	21.9	178.1

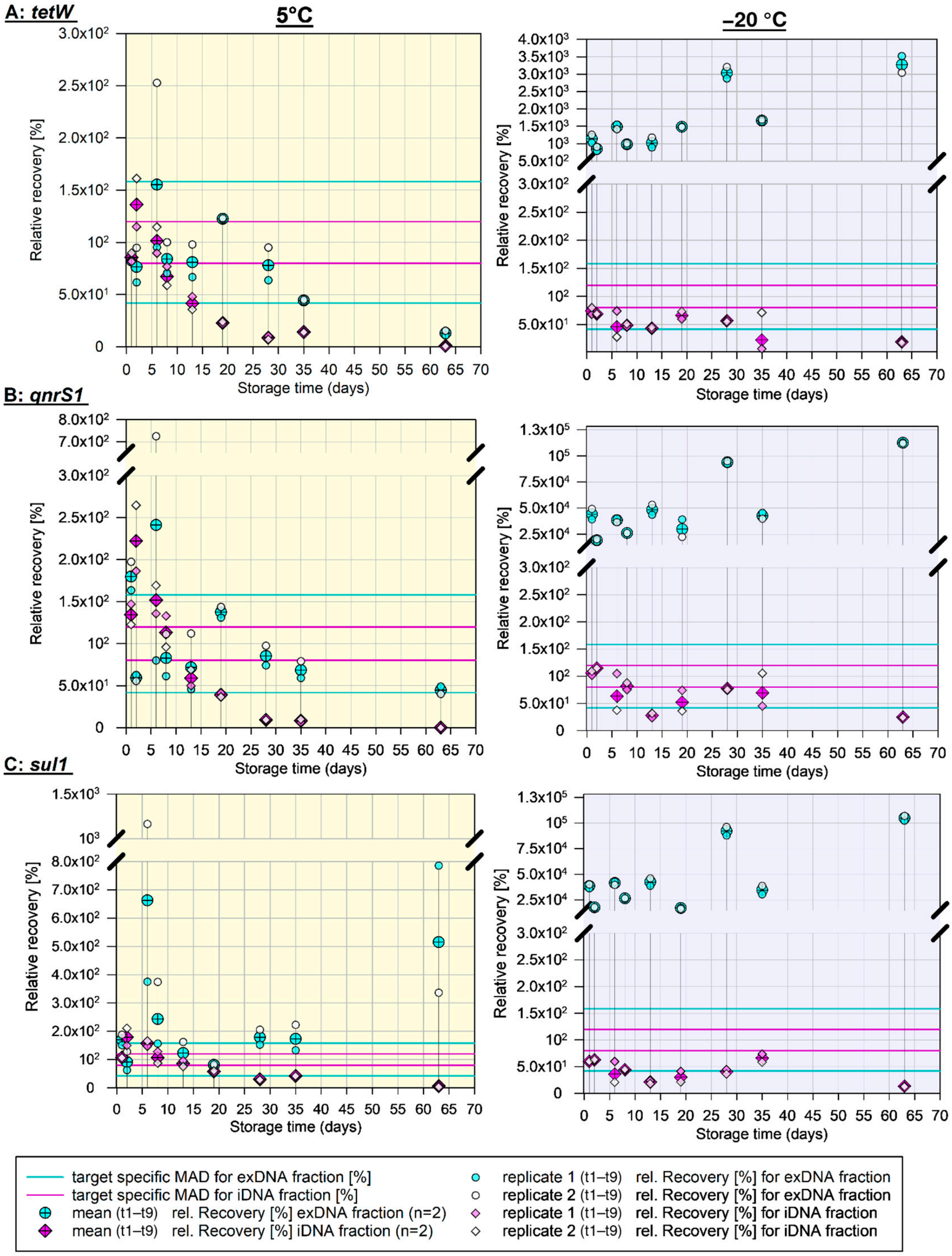

Figure S6: *Relative recovery of ARG concentrations in the iDNA-enriched and exDNA-enriched fractions during storage at 5 °C and −20 °C. The rows represent the ARG target genes sul1, tetW, and qnrS1, whilst the columns represent the two storage temperatures (yellow = 5 °C, purple = −20 °C). The relative recovery [%] was calculated for each observation as the ratio of the concentration at the storage time point t to the corresponding target- and fraction-specific mean of the concentration at t_0. Individual observations, mean at each time point are presented. The common t0 reference comprised replicates for iDNA (n=11) and for exDNA (n=10); t1-t9 each comprised two replicates per fraction and storage temperature. The purple (iDNA) and blue (exDNA) horizontal lines indicate the target- and fraction-specific limits for the maximum allowable deviation (MAD).*

References

1. Gao, M.; Kortum, P.; Oswald, F.L. Multi-Language Toolkit for the System Usability Scale. *International Journal of Human–Computer Interaction* **2020**, *36*, 1883–1901, doi:10.1080/10447318.2020.1801173.
2. Ahmed, W.; Smith, W.J.M.; Metcalfe, S.; Jackson, G.; Choi, P.M.; Morrison, M.; Field, D.; Gyawali, P.; Bivins, A.; Bibby, K.; et al. Comparison of RT-qPCR and RT-dPCR Platforms for the Trace Detection of SARS-CoV-2 RNA in Wastewater. *ACS ES T Water* **2022**, *2*, 1871–1880, doi:10.1021/acsestwater.1c00387.

.
